# Evaluation of genetic variation among Brazilian soybean cultivars through genome resequencing

**DOI:** 10.1186/s12864-016-2431-x

**Published:** 2016-02-13

**Authors:** João Vitor Maldonado dos Santos, Babu Valliyodan, Trupti Joshi, Saad M. Khan, Yang Liu, Juexin Wang, Tri D. Vuong, Marcelo Fernandes de Oliveira, Francismar Corrêa Marcelino-Guimarães, Dong Xu, Henry T. Nguyen, Ricardo Vilela Abdelnoor

**Affiliations:** Brazilian Corporation of Agricultural Research (Embrapa Soja), Carlos João Strass road, Warta County, PR Brazil; Londrina State University (UEL), Celso Garcia Cid Road, km 380, Londrina, PR Brazil; National Center for Soybean Biotechnology and Division of Plant Sciences, University of Missouri, Columbia, MO 65211 USA; Informatics Institute and Christopher S. Bond Life Sciences Center, University of Missouri, Columbia, MO 65211 USA; Department of Computer Science, University of Missouri, Columbia, MO 65211 USA

**Keywords:** *Glycine max*, Allelic variation, Genetic diversity, Positive selection, CNV

## Abstract

**Background:**

Soybean [*Glycine max* (L.) Merrill] is one of the most important legumes cultivated worldwide, and Brazil is one of the main producers of this crop. Since the sequencing of its reference genome, interest in structural and allelic variations of cultivated and wild soybean germplasm has grown. To investigate the genetics of the Brazilian soybean germplasm, we selected soybean cultivars based on the year of commercialization, geographical region and maturity group and resequenced their genomes.

**Results:**

We resequenced the genomes of 28 Brazilian soybean cultivars with an average genome coverage of 14.8X. A total of 5,835,185 single nucleotide polymorphisms (SNPs) and 1,329,844 InDels were identified across the 20 soybean chromosomes, with 541,762 SNPs, 98,922 InDels and 1,093 CNVs that were exclusive to the 28 Brazilian cultivars. In addition, 668 allelic variations of 327 genes were shared among all of the Brazilian cultivars, including genes related to DNA-dependent transcription-elongation, photosynthesis, ATP synthesis-coupled electron transport, cellular respiration, and precursors of metabolite generation and energy. A very homogeneous structure was also observed for the Brazilian soybean germplasm, and we observed 41 regions putatively influenced by positive selection. Finally, we detected 3,880 regions with copy-number variations (CNVs) that could help to explain the divergence among the accessions evaluated.

**Conclusions:**

The large number of allelic and structural variations identified in this study can be used in marker-assisted selection programs to detect unique SNPs for cultivar fingerprinting. The results presented here suggest that despite the diversification of modern Brazilian cultivars, the soybean germplasm remains very narrow because of the large number of genome regions that exhibit low diversity. These results emphasize the need to introduce new alleles to increase the genetic diversity of the Brazilian germplasm.

**Electronic supplementary material:**

The online version of this article (doi:10.1186/s12864-016-2431-x) contains supplementary material, which is available to authorized users.

## Background

Soybean [*Glycine max* (L.) Merrill] is considered one of the most important leguminous crops worldwide because of its use as human food, and in oil production. In Brazil, soybean became economically important in the 1970s, and since then, its significance in the world agricultural market has increased. Globally, Brazil is the second largest soybean producer, with 86.3 million tons harvested from 30.1 million hectares of cultivated area during the 2013-2014 growing season [1]. This clearly demonstrates the importance of this crop to Brazilian agribusiness and the strategic role of breeding programs focused on higher yield, stress tolerance, and crop quality.

However, soybean breeding in Brazil has a very recent history, with the first cultivar (cv.) developed in the 1940s. The success of soybean in Brazilian agribusiness is due to the direct results of increased production in traditional areas and the advancement of new agricultural frontiers, mainly in the Savannah region, associated with the availability of germplasm adapted to tropical regions [2]. Although soybean breeding programs in Brazil have led to progress and achievements, some factors continue to limit the crop production potential, including diseases and unfavorable environmental conditions. Indeed, the restricted nature of the Brazilian soybean germplasm increases the risk of new pathogenic pest variants or emerging diseases. In previous studies, Hiromoto and Vello [3] described 26 soybean ancestors with significant contributions to the Brazilian soybean germplasm. PI 548485 (Roanoke), PI 548445 (CNS), PI 548493 (Tokyo), and PI 548488 (S-100) are the most important ancestors, and a recent study showed that these four ancestors contributed to 55.3 % of the Brazilian soybean germplasm [4]. Moreover, the same study revealed six important ancestors of Brazilian soybeans that are shared with the U.S. soybean germplasm (CNS, S-100, Roanoke, Tokyo, PI 54610 and PI 548318), as the first Brazilian cultivars were developed based on the U.S. germplasm.

Therefore, the development of tools that support breeding programs to maintain the demand for cultivars with higher yields and that are adapted to different stress conditions is essential to meet the demand to feed a growing worldwide population. Techniques in genomics and molecular biology have emerged as important tools for advancing plant breeding with the goal of crop improvement, and new high-throughput sequencing platforms have arisen as alternative methods for trait discovery, allelic variation, and population studies as well and genome-wide association analysis (GWAS) in plants [5].

In soybeans, large-scale sequencing efforts have recently been realized with the first reference genome sequencing [6] of a 978-megabase (Mb) assembly of the Williams 82 cultivar, which allowed the identification of 46,430 genes distributed throughout 20 chromosomes. The same study showed that approximately 75 % of the genes in the soybean genome are present in multiple copies.

The wild soybean *Glycine soja* has also been studied at the genome level. Kim et al. [7] sequenced 915.5 Mb of a wild soybean accession and found 2.5 megabases of substituted sequences, 406 kilobases (kb) of InDels, 32.4 megabases of deletions and 8.3 megabases of new sequences when compared with the *Glycine max* reference genome cv. Williams 82.

Amidst the large amount of information generated by genome-wide sequencing, resequencing strategies have become important tools for studies of allelic variation. In other plant species, whole-genome resequencing has been widely used in various genomic studies, including *Arabidopsis* [8], corn [9], rice [10], cucumber [11] and sorghum [12]. In soybean, several resequencing efforts have also been reported recently. For example, by resequencing 31 wild and commercial soybean cultivars, Lam et al. [5] identified a high level of diversity in wild soybean accessions, which allowed the identification of 205,614 SNPs. Chung et al. [13] catalogued the genomic variation in commercial and wild soybean accessions from Korea and identified 3.87 million high-quality SNPs. In another study, Li et al. [14] analyzed the genome of 25 resequenced Chinese soybean accessions along with 30 soybean accessions identified in a public database and identified 5,102,244 SNPs and 707,969 InDels, of which 25.5 % had not been previously reported. Recently, 302 resequenced genomes of wild, landrace, and improved accessions of soybean were analyzed, and a total of 9,790,744 SNPs and 876,799 InDels were detected [15].

The large amount of sequence information continuously deposited in public databases demonstrates the value of such studies for a better understanding of the genetic basis of this leguminous crop. Furthermore, the advent of cost effective and new high-throughput sequencing technologies for genome-wide analysis have allowed deeper genome sequencing of a large number of lines of various crops. Thus, resequencing strategies are important tools for identifying variations that can be utilized in breeding programs for crops with limited genetic variation, such as soybean. The overall lack of available information increases the need for in-depth studies about the genomic diversity of the Brazilian germplasm. Moreover, resequencing analyses represent a powerful approach for identifying a large number of allelic/structural variations that can be useful for detecting important genes in breeding programs and for protecting soybean seed stock via cultivar fingerprinting.

In the present study, we resequenced 28 Brazilian soybean lines released over the last 50 years that are adapted to different regions in Brazil. These sequences were used to evaluate variations among the genomes throughout the history of Brazilian soybean breeding programs. Furthermore, we identified genomic regions associated with important variations, such as deletions, substitutions and duplications, which could be helpful for explaining divergence/similarity among different cultivars.

## Results and Discussion

### Sequencing and variation

**Fig. 1:**
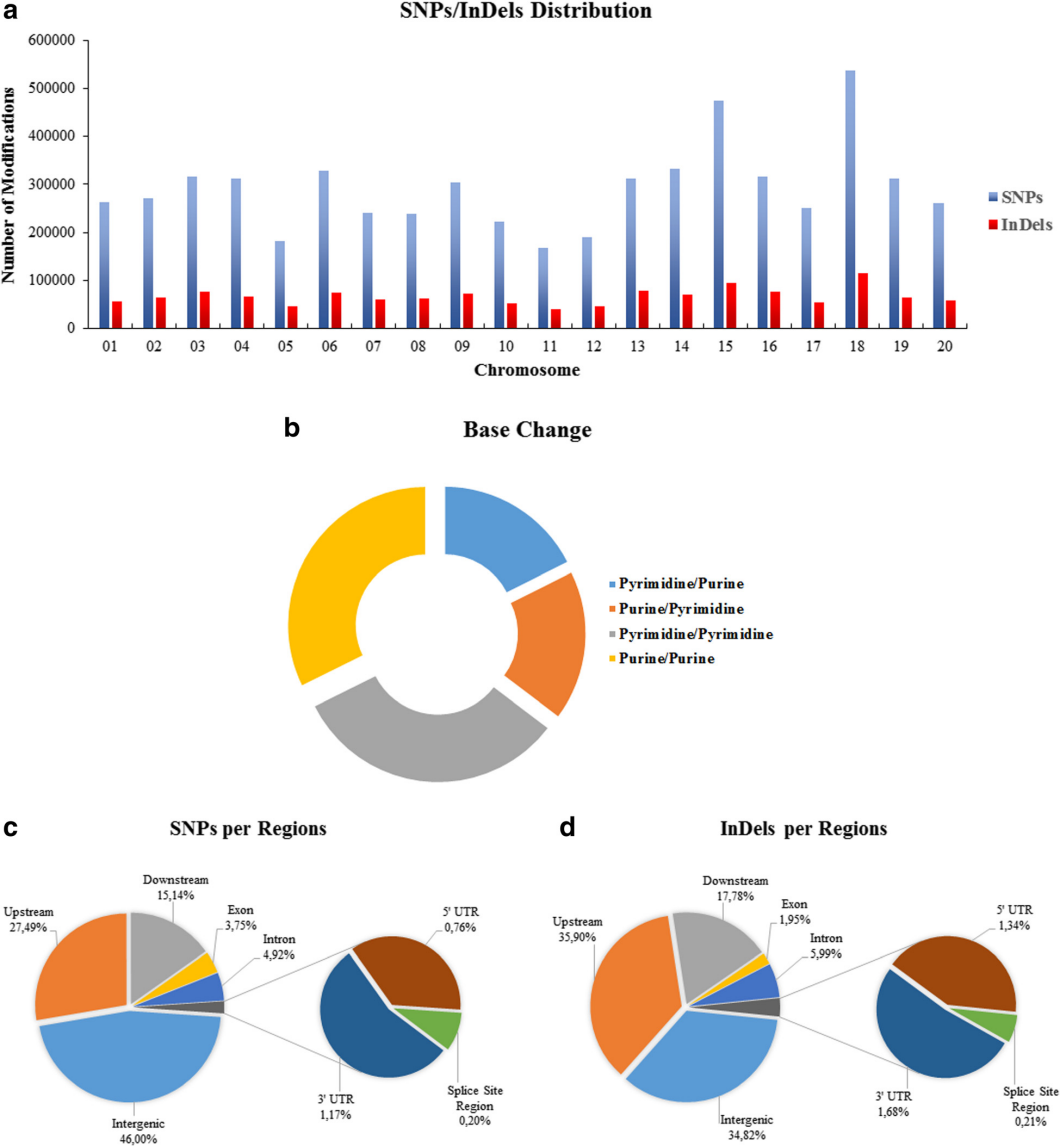
Summary of the major modifications caused by SNPs and InDels. **a** SNPs (blue) and InDels (*red*) distributed among the 20 soybean chromosomes. **b** Numbers of transition/transversion mutations: pyrimidine/purine (*blue*), purine/pyrimidine (*red*), pyrimidine/pyrimidine (*green*) and purine/purine (*purple*). **c** Percentage of SNPs per region in the soybean genome. **d** Percentage of InDels per region in the soybean genome

A total of 28 Brazilian soybean accessions were re-sequenced (Additional file 5: Table S1), resulting in the generation of approximately 5.5 billion paired-end reads with a read length of 100 bp and an average genome coverage of 14.8X. The percentage of reads mapped to the soybean genome for each accession was 94.3 %, which demonstrated that the resequencing effort covered most of the genome (Additional file 6: Table S2). When mapped against the reference genome, 5,835,185 SNPs were identified in the Brazilian lines, representing a higher value than previously reported [5, 13]. However, it was expected due to the high coverage depth in the present study compared with previous investigations. These SNPs are well distributed across all chromosomes, with chromosomes 15 and 18 demonstrating the largest number of SNPs (Fig. 1a) and the highest ratio of SNPs per chromosome length (Additional file 7: Table S3). As expected, most of the SNPs/InDels are homozygous. Nonetheless, 7.17 % of them are heterozygous, and the Embrapa 48 cultivar possesses the greatest number of heterozygous SNPs (Additional file 1: Figure S1a). When compared with the reference genome, most of the nucleotide changes can be classified as transitions, with a transition/transversion ratio (ts/tv ratio) of 1.83 (Fig. 1b). A total of 2,684,448 SNPs were detected in intergenic regions. In coding regions, we found a total of 218,671 SNPs in exons, 287,414 SNPs in introns and 112,790 SNPs in UTRs (Fig. 1c). The non-synonymous-to-synonymous ratio observed between the Brazilian accessions was 1.55. Although the ratio observed in this study was lower than those observed in other soybean studies [5], it is higher than that reported for other plants, such as sorghum [12] and rice [10]. The genomes of cvs. Santa Rosa and Doko have the highest number of SNPs, whereas cvs. BRS 284 and BRS/GO 8360 have the lowest numbers (Table 1).

**Table 1:** Total SNPs and InDels for each Brazilian soybean cultivar

Type	SNPs				InDels			
	Non-coding region	Coding region	None	Total	Non-coding region	Coding region	None	Total
**Anta 82**	848,752	5,231	99,327	953,310	203,207	25,458	1,390	230,055
**BR 16**	1,267,827	8,302	158,063	1,434,192	300,160	38,513	2,088	340,761
**BRS 232**	1,166,390	7,772	148,274	1,322,436	275,225	35,666	1,779	312,670
**BRS 284**	778,152	6,204	100,434	884,790	196,677	25,615	1,584	223,876
**BRS 360 RR**	1,078,635	7,169	128,689	1,214,493	252,198	32,059	1,731	285,988
**BRS 361**	1,017,791	5,595	115,310	1,138,696	222,364	27,243	1,354	250,961
**BRS Sambaiba**	1,318,067	8,113	162,389	1,488,569	311,172	39,236	1,909	352,317
**BRS Valiosa RR**	1,321,233	7,824	158,653	1,487,710	296,309	38,087	1,812	336,208
**BRS/GO 8360**	839,016	5,276	102,261	946,553	202,591	26,176	1,377	230,144
**BRS/GO 8660**	1,373,660	8,511	159,418	1,541,589	299,360	37,447	1,905	338,712
**BRS/GO Chapadões**	1,296,919	7,775	152,967	1,457,661	304,194	38,021	1,898	344,113
**BRSMG 850G RR**	1,273,571	7,868	157,019	1,438,458	301,255	38,665	1,891	341,811
**BRSMT Pintado**	1,326,229	8,574	151,052	1,485,855	299,477	36,717	2,006	338,200
**BRSMT Uirapuru**	1,376,297	8,346	162,189	1,546,832	314,856	39,732	1,909	356,497
**CD 201**	1,305,772	8,447	150,706	1,464,925	298,825	37,151	2,023	337,999
**Conquista**	1,338,601	7,955	159,887	1,506,443	317,096	39,952	1,952	359,000
**Doko**	1,414,796	9,372	165,725	1,589,893	327,783	40,606	2,221	370,610
**Embrapa 48**	1,091,441	7,882	136,232	1,235,555	264,083	33,767	1,807	299,657
**Emgopa 301**	1,208,853	7,825	144,216	1,360,894	281,240	35,661	1,758	318,659
**FT Abyara**	1,241,667	8,250	153,768	1,403,685	291,494	37,394	1,761	330,649
**FT Cristalina**	1,341,883	8,165	160,115	1,510,163	312,875	39,400	1,922	354,197
**IAC 8**	1,279,510	7,546	150,883	1,437,939	295,688	37,126	1,791	334,605
**IAS 5**	1,162,970	8,576	141,328	1,312,874	275,267	34,213	1,974	311,454
**NA 5909 RG**	949,130	6,184	108,680	1,063,994	222,730	27,813	1,398	251,941
**P98Y11**	1,341,733	8,376	156,262	1,506,371	301,238	37,248	1,942	340,428
**Paraná**	1,168,303	8,209	139,875	1,316,387	283,149	35,058	1,873	320,080
**Santa Rosa**	1,485,334	9,409	178,350	1,673,093	340,609	42,878	2,177	385,664
**VMAX RR**	1,008,968	6,154	107,398	1,122,520	231,321	27,871	1,396	260,588

**Non-coding regions:** corresponding to allelic variations up to 5 kb upstream or downstream of genes and intergenic regions modifications; **Coding region:** corresponding to UTR regions, exons, introns, and splice site modifications; **None:** no description available for the region

**Table 2:** Summary of regions under positive selection processes with F_ST_ and θπ values

Chromosome	Start	End	Number of SNPs	θπ (oldest cultivars)	θπ (newest cultivars)	F_ST_
07	40,100,001	40,110,000	41	0.00219	0.00000	0.7071
	40,110,001	40,120,000	12	0.00064	0.00000	0.7071
	40,140,001	40,150,000	26	0.00139	0.00000	0.7071
	40,150,001	40,160,000	31	0.00165	0.00013	0.7071
	40,160,001	40,170,000	36	0.00192	0.00006	0.7071
	40,630,001	40,640,000	21	0.00112	0.00000	0.7071
15	2,950,001	2,960,000	35	0.00187	0.00014	0.7071
	2,960,001	2,970,000	16	0.00085	0.00000	0.7071
17	3,010,000	3,020,000	17	0.00060	0.00000	0.8695
	3,030,001	3,040,000	23	0.00082	0.00000	0.8194
	3,040,001	3,050,000	41	0.00146	0.00002	0.8695
	3,050,001	3,060,000	13	0.00046	0.00000	0.7486
	5,560,001	5,570,000	76	0.00279	0.00000	0.8620
	5,570,001	5,580,000	31	0.00110	0.00000	0.8695
	5,580,001	5,590,000	26	0.00092	0.00000	0.8695
	5,610,001	5,620,000	22	0.00078	0.00000	0.8275
	5,620,001	5,630,000	34	0.00121	0.00000	0.8695
	5,660,001	5,670,000	39	0.00140	0.00000	0.8677
	5,670,001	5,680,000	26	0.00092	0.00000	0.8695
	5,680,001	5,690,000	35	0.00128	0.00000	0.8383
	5,710,001	5,720,000	28	0.00100	0.00003	0.8695
	5,730,001	5,740,000	20	0.00070	0.00000	0.8321
	5,740,001	5,750,000	45	0.00160	0.00004	0.8695
	5,750,001	5,760,000	26	0.00094	0.00000	0.8572
	5,760,001	5,770,000	74	0.00263	0.00000	0.8695
	5,770,001	5,780,000	24	0.00088	0.00001	0.8636
	5,780,001	5,790,000	39	0.00139	0.00000	0.8676
	5,790,001	5,800,000	25	0.00089	0.00000	0.8695
	5,800,001	5,810,000	63	0.00224	0.00000	0.8671
	5,810,001	5,820,000	50	0.00178	0.00000	0.8695
	5,820,001	5,830,000	48	0.00171	0.00000	0.8695
	5,830,001	5,840,000	48	0.00171	0.00003	0.8679
	5,840,001	5,850,000	27	0.00096	0.00000	0.8695
	5,850,001	5,860,000	24	0.00085	0.00007	0.8695
	5,860,001	5,870,000	69	0.00249	0.00010	0.8664
	5,870,001	5,880,000	32	0.00114	0.00000	0.8695
	5,880,001	5,890,000	66	0.00238	0.00000	0.8663
	5,890,001	5,900,000	76	0.00270	0.00003	0.8447
	5,900,001	5,910,000	58	0.00206	0.00000	0.8695
	5,910,001	5,920,000	14	0.00050	0.00007	0.8050
18	2,190,001	2,200,000	107	0.00571	0.00010	0.7032

**F**_**ST**:_ population fixation index coefficient; **θπ**: nucleotide diversity; **oldest cultivars**: Brazilian soybeans released before 1980; **newest cultivars**: Brazilian soybean cultivars released after 2000

A total of 1,329,844 InDels were detected among the Brazilian soybean accessions, lower than the proportion observed in other species [10, 12]. For InDels, the distribution along chromosomes and the homozygous/heterozygous proportion for each cultivar were similar to what was observed for SNPs (Figs. 1a and Additional file 1: Figure S1b). Approximately 463,106 of the InDels are in intergenic regions; 79,721 are in intronic regions, 40,105 in UTR regions and 25,861 in exons. Similar to the SNP analysis, Doko and Santa Rosa demonstrated the greatest number of InDels and BRS 284 and BRS/GO 8360 the lowest number. A summary of these variations is shown in Fig. 1d.

### Allelic variations in the Brazilian germplasm

The allelic variations found in the Brazilian germplasm have led to a large number of codon modifications in important genomic regions, and a large number of genes with allelic variations in Brazilian lines were revealed upon comparison with the soybean reference genome.

In all of the Brazilian lines, 21,263 loci share the same allelic variation that is divergent from the reference genome, of which 17,581 are SNPs and 3,682 are InDels. In addition to this information, 26,468 allelic variations, including 14,560 SNPs and 11,908 InDels, are shared among all of the Brazilian lines and are present in 19 U.S. accessions (Henry T. Nguyen laboratory, data not shown). One of the U.S. soybean accessions is of the cv. Williams 82 background, thereby increasing the chances of detecting the presence of sequencing errors in the reference genome or allelic variations exclusive to cv. Williams 82.

A total of 609 SNPs shared among all of the Brazilian cultivars were identified in important regions of 303 genes (Additional file 8: Table S4). According to SoyBase enrichment analysis [16], 34 genes are associated with the generation of metabolite precursors and energy related to DNA-dependent transcription/elongation and processes related to photosynthesis. Some of these processes can also be related to cell respiration and ATP synthesis-coupled electron transport (Additional file 9: Table S5).

A similar analysis of non-synonymous mutations in important gene regions was conducted to identify InDels exclusive to the Brazilian cultivars (Additional file 10: Table S6). A total of 59 InDels are non-synonymous modifications detected in 52 genes, most of which are in exons; an exception is one haloacid dehalogenase-like hydrolase gene (*Glyma.04G110000*) that contains a frameshift modification associated with the loss of a stop codon.

In addition, we found seven genes with putative modifications due to the presence of SNPs resulting in the loss of a start codon shared among all of the Brazilian lines. These genes are related to protein binding (*Glyma.07 g153200*), ATP synthesis-coupled electron transport and NADH dehydrogenase (ubiquinone) activity (*Glyma.15 g246000*) and include three putative pseudogenes on chromosome 16 (15,19-16,88 Mb): *Glyma.16 g017300* (serine/threonine protein kinase), *Glyma.16 g019100* (proprotein convertase subtilisin/kexin) and *Glyma.16 g019200* (S1/P1 nuclease related to DNA catabolic processes). We identified six SNPs in stop codons, but only two of the genes have been annotated: *Glyma.07 g156200* has an AP2 domain related to transcriptional regulation, and *Glyma.18 g132800* is associated with ATP binding because it is a cell component of chloroplasts.

Moreover, we detected four SNPs that lead to alternative splicing, including a gene with a PPR repeat domain (*Glyma.18 g056000*), which could be related to plant disease resistance mechanisms, NADH-ubiquinone/plastoquinone (*Glyma.10 g068800*), and DNA replication protein (*Glyma.16 g005600*). No annotation was found for *Glyma.17 g186300*.

Finally, we identified putative exon losses in the Brazilian cultivars due to the presence of InDels in three genes. BRS Sambaiba has an InDel that is responsible for the loss of the second exon of *Glyma.09G159600*, a sodium/calcium exchanger protein. A similar loss was observed in the first exon of 1,3-beta-glucan synthase (*Glyma.08G308200*) in cvs. BR 16 and Embrapa 48. In addition, the first exon of *Glyma.18G128800* is absent in cvs. BRSMT Uirapuru, CD 201, Emgopa 301, FT Abyara and FT Cristalina. Compared with the reference genome, a heterozygous InDel is present in cvs. BRSMT Uirapuru, FT Abyara and FT Cristalina.

Several gene modifications were found in the Brazilian accessions compared with the reference genome. Once confirmed, these differences could provide insight into plant adaptation to the tropical conditions in Brazil as well as the loss of function of genes that may not have a key role in survival. However, more detailed studies are needed to verify the functions of these modified genes, especially those related to photosynthesis and the generation of metabolites, precursor metabolites and energy processes.

### Influence of allelic variation in determinate/indeterminate growth habits and maturity group distribution

A total of 96 SNPs and 32 InDels were detected in the *E1*, *E2*, *E3*, and *E4* loci. Most of the allelic variation was observed in the non-coding gene region, including 75 allelic modifications 5 kb upstream of the loci; 76 allelic modifications were detected in coding regions, mostly in introns. Furthermore, three non-synonymous modifications in exons and one in a splice site region were found. We observed new allelic variations in these loci, as well as variations similar to those already described [17–22]. According to a previous study, cv. Williams 82 has the genotype “e1-as, E2, E3, E4” [17]. Among the Brazilian cultivars, BRS/GO Chapadões, BRSMG 850G RR, Conquista, BRS Valiosa RR, VMAX RR, and NA 5909 RG exhibit a dominant genotype for all loci according to allelic comparisons with Williams 82. In contrast, based on an allelic variation comparison with Williams 82, BRS 361 is the only accession with a recessive genotype, excluding the *E4* locus.

**Fig. 2:**
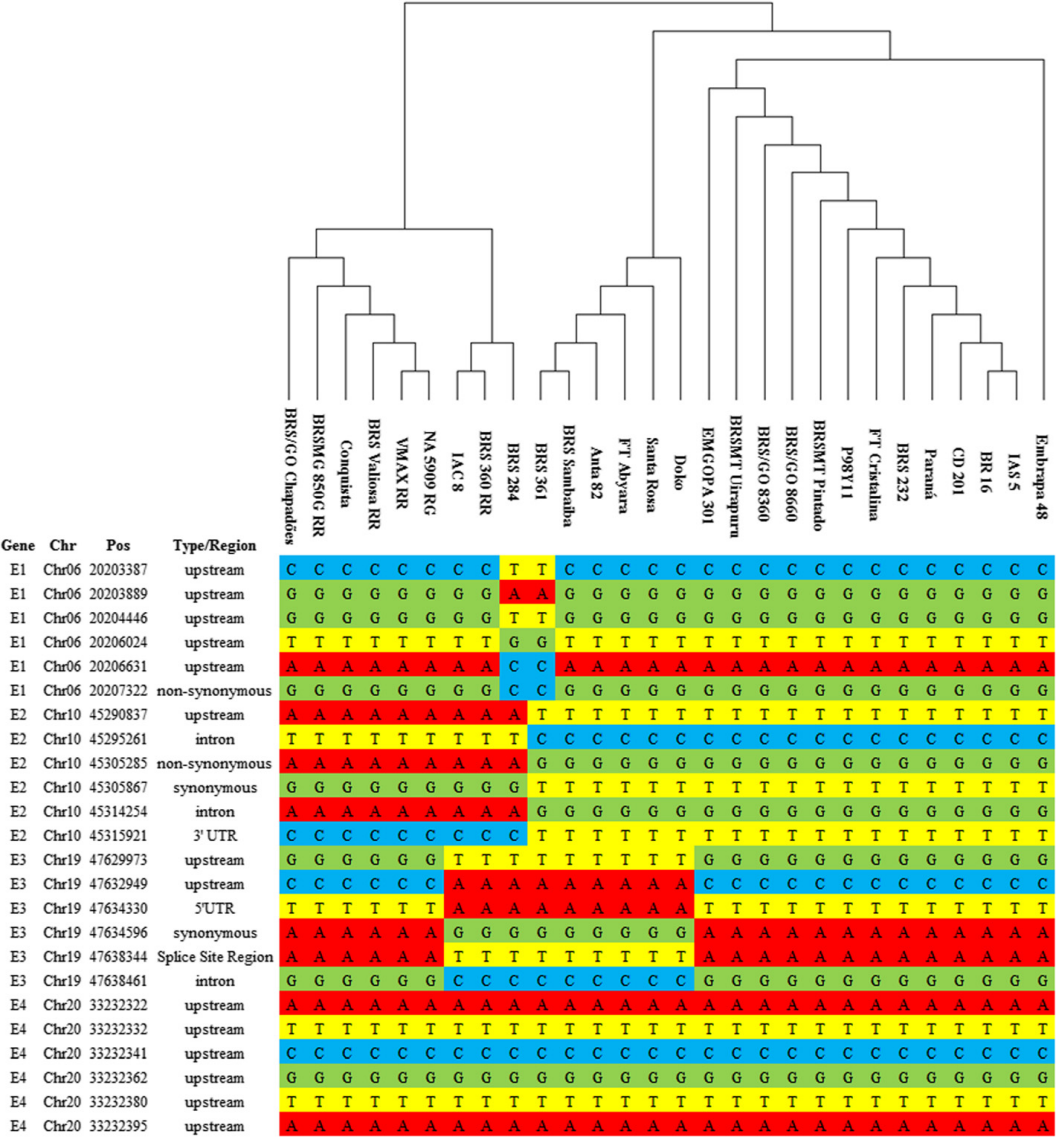
Twenty-four SNPs identified in *E1-E3* loci, and the regulatory region of the *E4* gene. **Upstream:** SNPs were detected up to 5 kb upstream of the coding region; **Non-synonymous:** SNP variants causing a codon that produces a different amino acid; **Intron:** SNPs detected inside an intron; **3’ UTR:** SNPs found in the 3’UTR; **5’UTR:** SNPs was found in the 5’ UTR; **Splice Site Region:** sequence variants in which a change has occurred within the region of the splice site, either within 1-3 bases of the exon or 3-8 bases of the intron

Our results clustered some cultivars according to their relative maturity group (RMG) (Fig. 2), and all of the southern determinate soybeans clustered together; similar clustering was observed for most of the northern soybeans. However, some accessions with a high RMG that clustered closely to those with a low RMG. This result suggested that other *E* locus have had an important impact on the adaptability of cultivars in Brazil.

Cultivars BRS 284 and BRS 361, both indeterminate soybeans with great adaptability, possess allele e1, as observed in Williams 82 for the *E1* locus, and a similar haplotype. For the *E2* locus, 19 cultivars showed alleles different from those in the reference genome, including all of the determinate soybeans from South Brazil. Williams 82 has the dominant *E2* allele, indicating that the 19 cultivars harbor allelic variations that might influence the function of this locus. At the *E3* locus, we observed a recessive allele in four cultivars released before 1990 and in five cultivars released after 2000, including four with semi-determinate/indeterminate growth.

None of the Brazilian cultivars carry allelic variations in the coding region of the *E4* locus, showing the same genotype as that of Williams 82. However, a 3.61 - 3.69 kb interval upstream of the locus region contains 17 allelic variations that are shared among all of the Brazilian cultivars but differ from Williams 82. This finding suggests that a regulatory region may influence the *E4* locus in Brazilian cultivars. According to a previous study, modifications in the *E4* locus play a key role in adaptations to high-latitude environments [21], and the *E3* and *E4* loci have a role in pre- and post-flowering development in soybeans [23]. Thus, our results indicate that modifications in the regulatory *E4* locus may have served crucial functions in the adaptation of all cultivars in Brazil.

We identified non-synonymous modifications in *E1*, *E2*, and *E3* exons, with the *E3* modification identified only in cv. Doko. Moreover, one SNP was identified close to a splice site of the *E3*. For *E4*, we only detected modifications in an interval between 3,610 and 3,696 bp upstream of the locus, which might represent modifications in a regulatory region.

A similar analysis was performed for the plant growth habit *Dt1*: a total of 56 SNPs and 10 InDels were identified in this locus. As in the case of *E* loci, the number of allelic variations in non-coding regions was higher compared to coding regions; in fact, only one non-synonymous SNP was found in an exon in the present analysis. Importantly, the allelic variation identified in this study was able to distinguish all of the cultivars according to their growth habit (Additional file 2: Figure S2). Williams 82 has an indeterminate grown habit, indicating the presence of a dominant allele for the *Dt1* locus [24]. According to our sequencing data, Anta 82, BRS 284, BRS 360 RR, BRS 361, BRS/GO 8360, NA 5909 RG, and VMAX RR have allelic variations that are similar to Williams 82, suggesting that they also carry the dominant allele *Dt1*.

*Dt1* locus analysis clearly grouped all of the accessions with determinate growth, with a non-synonymous SNP identified in this locus in nearly all of the cultivars with determinate growth, excluding cv. Doko. As this finding has been reported in other studies, this SNP appears to be important for the function of Dt1, which indicates that the growth characteristics associated with this allele, might be affected in cv. Doko. Because cv. Anta 82 is semi-determinate, a depth analysis of the *Dt2* locus is necessary to confirm our findings because the two loci (*Dt1* and *Dt2*) with epistatic interactions control semi-determinate plants. Soybeans with the genotype “Dt1, dt2” are indeterminate plants, in contrast to semi-determinate plants of the “Dt1, Dt2” genotype [24], which suggests that the allelic variation in *Dt1* is able to clearly cluster cultivars according to their genotype.

Finally, Anta 82, BRS 284, and BRS 361 carry allelic modifications in the *E3* and *Dt1* as well as in a regulatory region of the *E4*. According to a previous study, *E3* and *E4* have a meaningful effect on the up-regulation of *Dt1* expression in plants [23, 25], and our findings might help in understanding the great adaptability capacity of these cultivars to different RMG.

### Low divergence in the Brazilian soybean germplasm

**Fig. 3:**
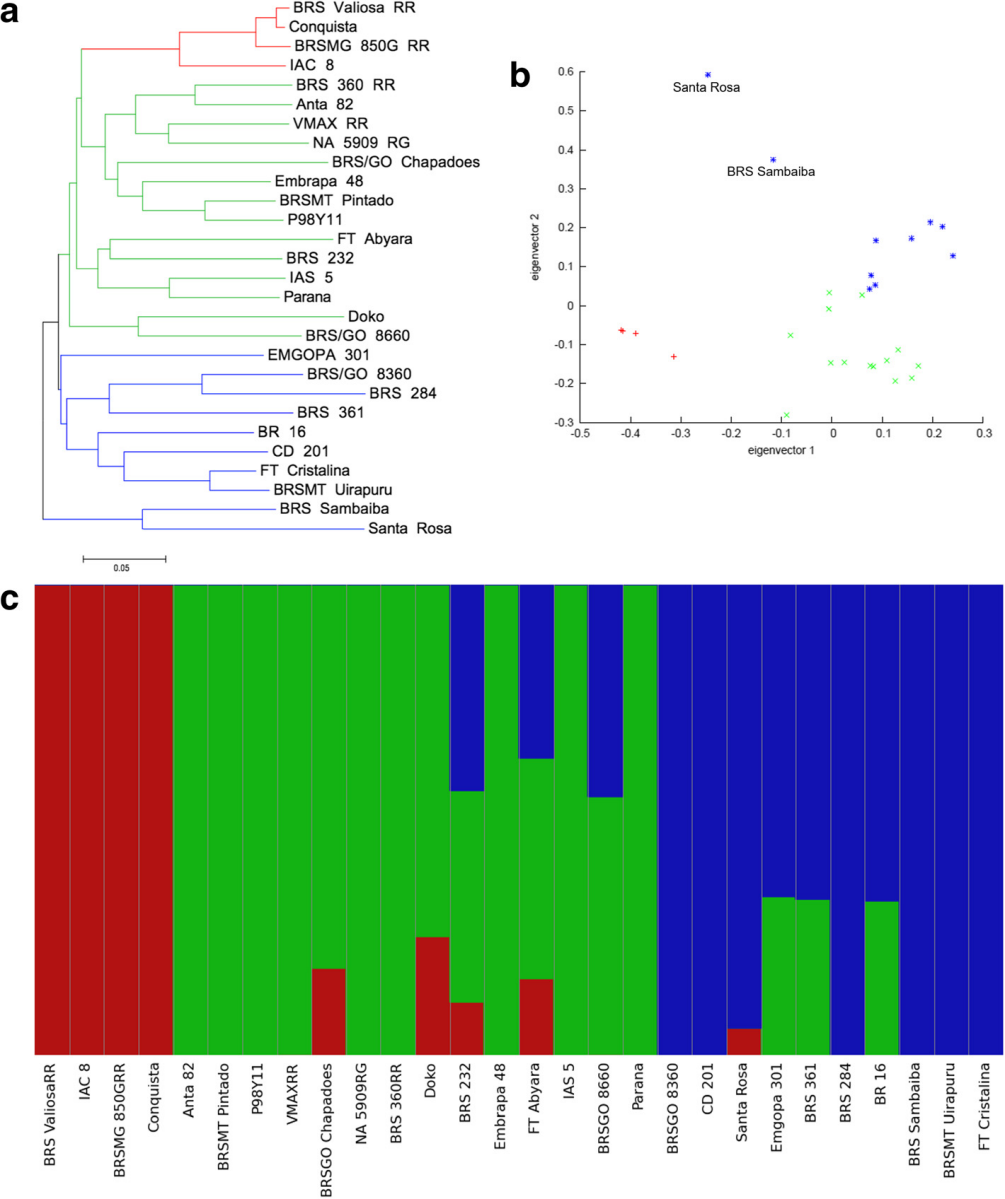
Population structure analysis of 28 Brazilian soybean cultivars. **a** Neighbor-joining phylogenetic tree generated for the 28 Brazilian soybean accessions. **b** Principal Component Analysis (PCA) from the 28 Brazilian soybean cultivars, **c** Bayesian clustering (FastStructure, *K* = 3) for the 28 Brazilian soybean cultivars

Brazilian soybean germplasm has a very narrow genetic diversity due to a very recent breeding program history and the presence of a small number of ancestors that are mainly derived from U.S. soybean germplasm. To study the population structure of the Brazilian soybean germplasm, we constructed a neighbor-joining (NJ) tree based on sequencing data for the Brazilian soybean cultivars (Fig. 3a) in which the accessions were grouped according to their genealogy.

Moreover, the seven cultivars harboring dominant alleles for the *Dt1* locus clustered closely together. This finding suggests an influence of the stem growth habit on the clustering of Brazilian cultivars and confirms our results of *Dt1* locus allelic variation. In addition, some soybean cyst nematode-resistant cultivars clustered closely together. BRSMT Pintado, P98Y11, and BRS/Chapadões display the Peking-type resistance and Anta 82 and VMAX RR the PI088788-type resistance. BRS 360 RR is a susceptible cultivar that carries a Peking-type *Rhg1* but no *Rhg4* locus in its genome, suggesting the presence and influence of the *Rhg1* locus in this germplasm clustering.

The principal component analysis (PCA) and the genetic structure of the collection of Brazilian soybean lines used were examined in this study. The PCA provided similar results from the NJ tree, with the Brazilian accessions clearly separated into three groups (Fig. 3b). Furthermore, the results obtained in PCA highlighted that Santa Rosa and BRS Sambaiba are in the same cluster, as we observed in NJ tree, but they are not so much close due some genetic divergence among the cultivars.

The *K* value was established as ranging between 1 to 10, and the best model components used to explain the structure for these data was model *K* = 3. The structure bar plot showed similar results observed in the NJ tree and PCA, with most of the accessions clustered according to their background (Fig. 3c). Moreover, some evidence for admixture was observed for BR 16, BRS 232, BRS 361, BRS/GO 8660, BRS/GO Chapadões, Doko, EMGOPA 301, FT Abyara and Santa Rosa. The results suggest that the Brazilian soybean germplasm is still very homogeneous, with potential introgression in a few cultivars. Evaluating 435 cultivars and 27 SSR markers, Priolli et al [26] was able to cluster Brazilian soybeans into two groups (*K* = 2). The discrepancy in *K* between the present and previous studies is mainly due to the number of cultivars and markers used in our study. A small number of accessions associated with a large number of SNPs has been used in other studies to clearly separate some cultivars and wild soybeans [5, 13].

Breeding programs always focus on the development of cultivars with the best performance under the influence of various environmental and field conditions. Thus, the development of cultivars tends to select and consequently modify certain genes/QTLs over time by increasing/removing important alleles in the selected germplasm. Accordingly, the identification of regions with high diversity as well as those with a low level of modification is extremely important for improving soybean adaptation to various environmental conditions in breeding programs.

To identify genomic regions with high levels of diversity between old and more recent cultivars, we calculated the fixation index (F_ST_) among the Brazilian accessions. Regions with high F_ST_ values could be related to artificial selection events, and regions with low F_ST_ values could indicate the existence of little genetic differentiation between accessions.

We identified 998 10-kb regions with F_ST_ values higher than 0.45 distributed in most of the soybean chromosomes. Chromosome 16 has the highest number of sub-regions with high F_ST_ values. Two chromosomes, 9 and 13, present no sub-regions with high F_ST_ values, which may be because these chromosomes do not have a strong influence on artificial selection during the development of new cultivars.

In contrast, we detected 2,097 sub-regions with F_ST_ values lower than 0.02, which revealed a large number of genomic regions with low diversity between the latest and oldest cultivars. Chromosome 6 contains the greatest number of these sub-regions with low diversity, with chromosome 16 having the smallest number. Lam et al [5] identified 369 sub-regions with high *F*_ST_ values and 101 sub-regions with low F_ST_ values in a comparison between wild and commercial soybeans, and the proportion of high/low F_ST_ values detected was higher compared with our results. This finding can be explained by more divergent data because two different species, *Glycine soja* and *Glycine max*, were compared in that previous study; conversely, only commercial *G. max* accessions from the same geographic region were used in our study. A large number of sub-regions with a low level of diversity demonstrate that the Brazilian soybean germplasm has remained narrow. These observations are consistent with the results of previous studies in which 444 Brazilian soybean lines displayed the same pattern [4], whereby a cumulative relative genetic contribution of 57.6 % was attributed to only four main ancestors, with an increase in the number of ancestors in the germplasm over time.

### Regions affected by positive selection processes in the Brazilian germplasm

Forty-one sub-regions with high F_ST_ values associated with a low level of nucleotide diversity (θπ) were identified on chromosomes 7, 15, 17, and 18 in the new cultivars compared with the old cultivars (Table 2). This number is lower than that reported by Zhou et al. [15], who identified 230 100-kb regions using a selective sweep. The main reason for this discrepancy is the large number of accessions used in that study compared with our study. Furthermore, the previous analysis was conducted with wild, landrace and improved soybeans, whereas only Brazilian cultivars were examined in our study.

**Table 3:** Number of unique SNPs, InDels and CNVs for each Brazilian soybean cultivar

Name	SNPs	InDels		Total	CNVs		Total
		Deletion	Insertion		Deletion	Insertion	
**Anta 82**	3,586	471	462	933	11	27	38
**BR 16**	7,036	881	796	1,677	4	7	11
**BRS 232**	3,653	482	388	870	35	18	53
**BRS 284**	62,279	4,224	4127	8,351	100	63	163
**BRS 360 RR**	3,731	588	541	1,129	22	4	26
**BRS 361**	10,778	1,130	946	2,076	10	53	63
**BRS/GO 8360**	5,328	775	654	1,429	8	43	51
**BRS/GO 8660**	20,388	1768	1,489	3,257	21	2	23
**BRS/GO Chapadões**	74,314	7,651	7,438	15,089	23	9	32
**BRSMG 850G RR**	318	81	57	138	4	6	10
**BRSMT Pintado**	3,116	391	350	744	12	3	15
**BRSMT Uirapuru**	10,662	1,069	927	1,996	6	9	15
**BRS Sambaíba**	31,811	3,237	2,791	6,028	23	5	28
**BRS Valiosa RR**	344	101	58	159	5	1	6
**CD 201**	11,050	1,277	1,098	2,375	18	9	27
**Conquista**	1,486	200	174	376	3	2	5
**Doko**	42,826	4,287	3,785	8,071	32	25	57
**Embrapa 48**	1,882	253	234	487	15	17	32
**Emgopa 301**	12,590	1,487	1,210	2,697	8	10	18
**FT Abyara**	36,447	3,920	3,685	7,605	20	10	30
**FT Cristalina**	458	102	76	178	3	3	6
**IAC 8**	41,325	2,973	2,637	5,610	25	8	33
**IAS 5**	8,918	1,195	1,110	2,305	37	103	140
**NA 5909 RG**	22,691	2,504	2,121	4,625	29	19	48
**P98Y11**	18,590	1,538	1,342	2,880	32	30	62
**Parana**	6,835	626	466	1,094	11	5	16
**Santa Rosa**	96,105	8,324	7,602	15,926	48	9	57
**VMAX RR**	3,215	423	400	823	6	22	28
**Total**	**541,762**	**51,958**	**46,964**	**98,928**	**571**	**522**	**1,093**

**Fig. 4:**
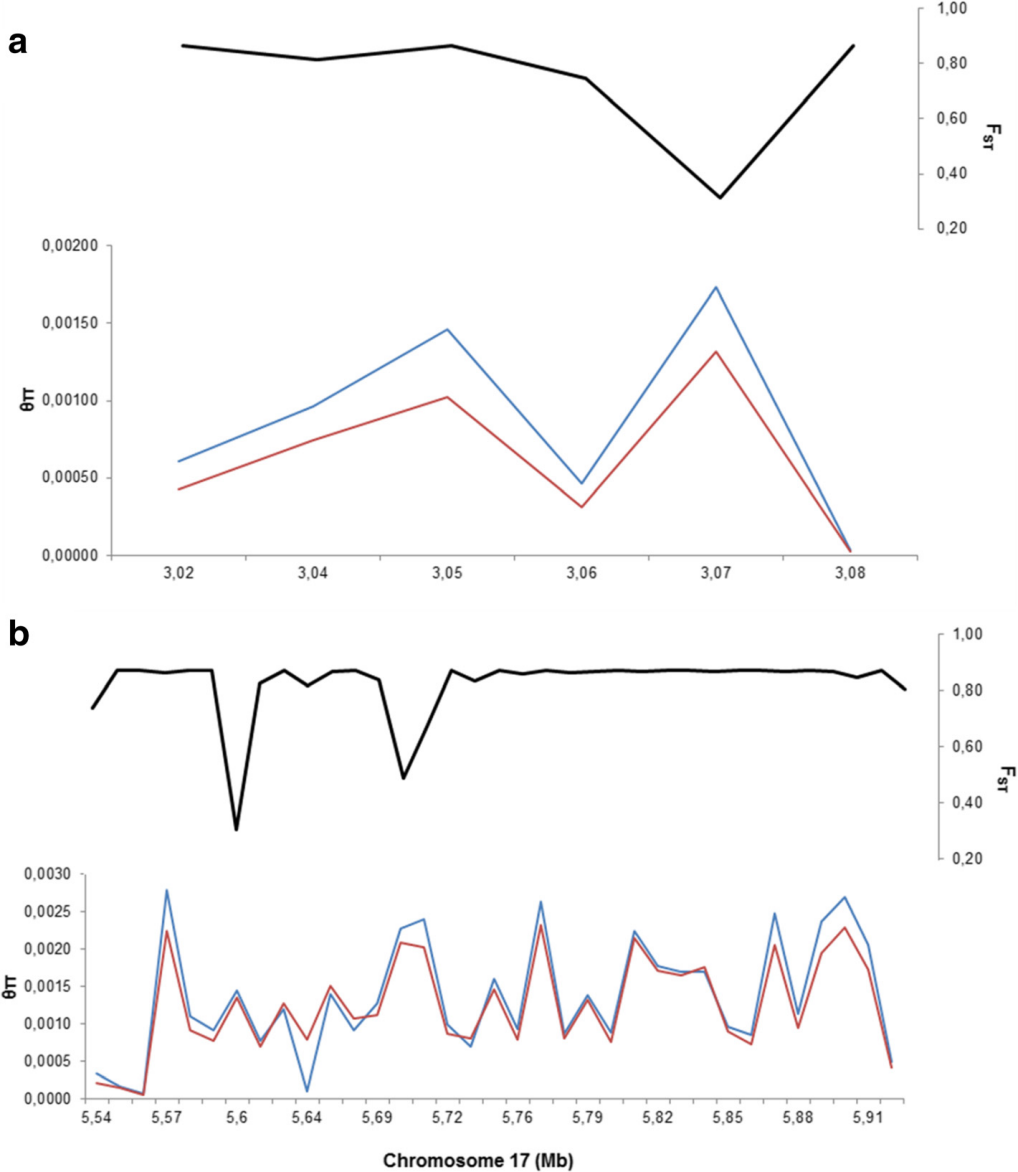
Two regions between 3.01-3.09 Mb (**a**) and 5.53-5.92 Mb (**b**) on chromosome 17 under positive selection. The red line corresponds to the nucleotide diversity of the newest cultivars and the blue line the oldest cultivars. The black line is the F_ST_ values between the oldest and newest cultivars

We identified 32 sub-regions with a size of 10 kb within two intervals on chromosome 17; four sub-regions within the 3.01-3.06 Mb interval with 100 SNPs and 28 sub-regions between the 5.56-5.92 Mb interval with 1,150 SNPs (Fig. 4). Most of the SNPs identified in both intervals were able to differentiate Doko, IAC 8, IAS 5 and Paraná from the other cultivars. These intervals have been previously described in other studies due to the presence of a large number of QTLs, such as those related to seed size [27–30], seed genistein/palmitic acid content [31, 32], plant/root weight, phosphorus content [33], canopy wilting [34], and resistance to soybean cyst nematodes [35] and white mold [36].

Furthermore, we identified additional sub-regions with high F_ST_ values on chromosomes 7, 15, and 18. Six sub-regions located at the end of chromosome 7 were detected, and all of these sub-regions carry SNPs that reveal a difference between cultivars IAC 8, Santa Rosa, and Doko compared with the other cultivars. Tajuddin et al [37] described two QTLs for seed oil content within these sub-regions. In the present study, we identified four genes between 40.10-40.17 Mb on chromosome 7: *Glyma.07G223900* (DNA helicase PIF1/RRM3, associated with telomere maintenance), *Glyma.07G224100* (gene with a B3 DNA-binding domain), *Glyma.07G224400* (NusB family associated with the regulation of transcription) and *Glyma.07G224600* (glucosidase 2 subunit beta). However, three other sub-regions detected on chromosome 15 (2.95-2.97 Mb with 51 SNPs) and 18 (2.19-2.20 Mb with 107 SNPs) are located at the beginning of these chromosomes. Only on chromosome 18 did we identify a modified gene due to the presence of an SNP: *Glyma.18G029000*, an amino acid transporter. However, several studies have reported the presence of QTLs that control important traits in these sub-regions. Indeed, several QTLs responsible for seed volume/length [27], isoflavone content [38], oleic/linoleic acid content [39] and protein/oil content [37, 40] have been identified on chromosome 15, and most of the identified QTLs on chromosome 18 are related to soybean cyst nematode resistance [41–48] and protein content [49]. The SNPs on chromosome 15 found in this study differentiated IAC 8, Paraná, and Doko from the new cultivars; however, we identified a similar pattern in cv. Embrapa 48 compared with the oldest cultivars, which could be explained by the presence of Paraná in its pedigree. Furthermore, the SNPs on chromosome 18 identified in the present study differentiated IAS 5, Paraná, and Doko from the most recent cultivars.

The high F_ST_ values associated with high *θπ* values in the oldest compared with the most recent cultivars confirmed the presence of sub-regions under positive selection processes. Thus, the Brazilian accessions experienced meaningful modifications in these 41 sub-regions over time. The presence of important traits within these sub-regions associated with a large difference in Brazilian soybean production over time and high F_ST_ values reinforce the notion of the existence of sub-regions that were influenced by positive selection.

We also identified a large number of regions with F_ST_ values less than 0.02. This result suggested the presence of regions with low diversity, indicating the presence of balancing selection. A portion of these regions under balancing selection could have important genes/QTLs that are responsible for survival. This finding, together with the detection of a large number of regions with high FST values, could be an important target for breeding programs to maintain these regions under positive selection. Moreover, the identification of regions under balancing selection that are not related to essential plant processes could be another important target for the insertion new alleles that could improve major traits in Brazilian cultivars.

### Copy number variations could explain the observed divergence among cultivars

CNVs refer to structural modifications that result in changes in copy number in a specific region of the genome. Such modifications may vary in size, and recently some studies have demonstrated their broad importance because they are linked to several traits, including some diseases in humans such as Alzheimer’s disease [50], autism [51] and Parkinson’s disease [52]. In soybean, a significant number of CNVs are associated with important traits, such as resistance to cyst nematode [53] and hilum color [15]. Moreover, a total of 162 CNVs have been identified as being potentially selected during soybean domestication and improvement processes [15]. As the identification of these CNVs in the soybean genome is extremely important, we analyzed all of the Brazilian soybean lines to identify important CNVs related to the divergence that has accumulated during the time between the oldest and the most recent accessions.

A total of 3,880 sub-regions containing CNVs across 20 chromosomes were detected in the Brazilian lines. The greatest number of CNV regions was identified on chromosomes 14 and 17, and the lowest number was found on chromosome 16. A summary of the number of CNVs detected for the cultivars is shown in Additional file 3: Figure S3.

**Fig. 5:**
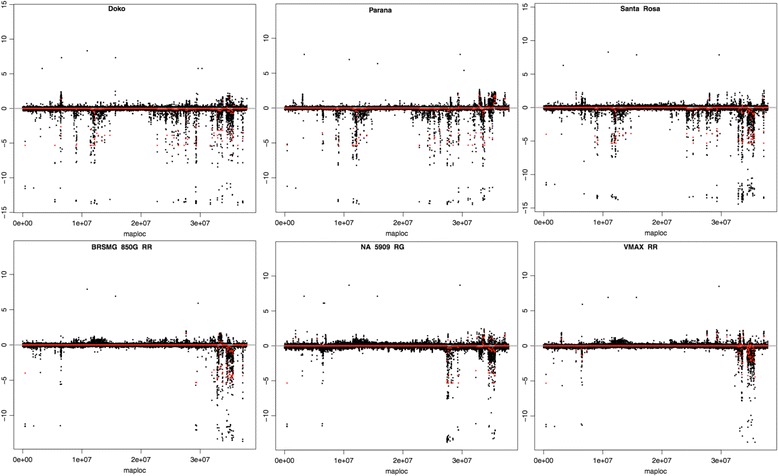
Copy number variations detected on chromosome 16 for the oldest and newest Brazilian cultivars. The x-axis represents the genomic position and y-axis the log-ratio of the read counts. The red dots are the copy number call of each segment

When comparing the oldest to the most recent cultivars, chromosome 16 shows CNVs in 12 sub-regions (Fig. 5). More than 80 % of the most recent cultivars do not have these deletions, which were only present in the oldest cultivars, Doko, EMGOPA 301, FT Abyara, IAS 5, Paraná, and Santa Rosa. One of these regions, ranging from 26.20-26.21 Mb, was not found in any cultivar developed after 2000. Furthermore, this CNV is not present in more than 70 % of the accessions prior to 1999. These results suggest that the 12 sub-regions identified on chromosome 16, especially the one described most recently, were acquired more recently in the breeding process. Other studies have described QTLs associated with flowering and maturity [54, 55], pod number and quality [56, 57], and leaflet format [58] in these intervals, increasing the possibility of the influence of CNVs on modifications over time.

Other important CNV regions that distinguished the oldest soybean lines from the most recent ones were detected on chromosomes 6, 7, 8, 9, 13, 15, and 17 (Additional file 4: Figure S4). Five meaningful deleted regions shared among more than 70 % of the most recent cultivars were detected on chromosome 15 between 41.37-42.68 Mb. Cultivars IAC 8, IAS 5, Paraná, Santa Rosa, Doko, and FT-Abyara show common insertions for four CNV regions, and six additional accessions carry these insertions: BR-16, MG/BR46, BRS 232, BRS Sambaíba, BRS Valiosa RR and BRMG 850G RR. These lines share a common ancestry with the oldest accession examined herein, which could explain the presence of the regions in these accessions. These patterns could indicate the presence of a duplicated region in the oldest cultivars and a deletion in the most recent cultivars.

Furthermore, relevant results were obtained for chromosome 7. Five sub-regions between 11.60-12.44 Mb exhibit deletions only present in the oldest cultivars, Doko, IAS 5, and Paraná, and in the four most recent cultivars, BRS 361, BRS/GO 8660, BRS/GO Chapadões and VMAX RR. Moreover, a deletion identified between 40.60-40.62 Mb was detected only in cvs. Doko, Santa Rosa, and IAC 8. All of the accessions produced during the 1981-2000 period lack this last CNV, potentially suggesting that this sub-region has been introgressed into the Brazilian soybean germplasm by 1980. Some studies have suggested the presence of QTLs related to yield [59], plant height [60], and oleic acid content [61] in this interval, and such introgressions could be important for soybean adaptability and productivity in Brazil.

We also identified important deletions on chromosomes 6, 9, and 13 in the oldest accessions and in a few recent lines. Three deletions on chromosome 6 were found in Doko, IAC 8, Paraná, and Conquista and three in the most recent lines, Anta 82, BRS Valiosa RR and BRSMG 850G RR. Cultivar CD 201 displays an insertion in the same region. Thus, more than 78 % of the Brazilian accessions produced after the 1970s demonstrate introgression of these three regions in their genomes over time. Chromosome 9 shows a deletion of 8 kb in CD 201, IAS 5, Paraná, Santa Rosa but in less than 30 % of the most recent cultivars, and only four recent lines (Anta 82, BRS 232, BRS/GO 8360, and BRS Sambaíba) exhibit the same pattern as the oldest cultivars. Thus, it is possible that these sub-regions were introgressed in the majority of the most recent accessions, excluding the CNVs we identified. Finally, chromosome 13 exhibits deletions in the oldest cultivars, Doko, IAC 8, IAC 5, and Paraná. This finding could indicate the presence of introgression in soybeans produced after the 1970s, and the presence of a QTL in another study associated with productivity [62] revealed the importance of this CNV.

Overall, the CNV analysis demonstrated that it is an important tool for verifying meaningful modifications in genomes. Indeed, the detection of this modified region will greatly impact future genomic studies in soybean regarding such aspects as the importance of gains/losses of these regions in QTL and genes.

### Application of unique allelic and structural variations to cultivar fingerprinting

We identified exclusive allelic and structural variations for each of the lines used in our study. A total of 541,762 SNPs, 98,922 InDels and 1,093 CNVs exclusive to each cultivar were detected (Table 3).

The results showing some cultivars with a large number (more than 40,000) of exclusive variations yet others with very few (less than 1,000) can be explained by the small number of cultivars used in this study. As expected, BRS Valiosa RR and Conquista showed very little exclusive variation because they are very closely related; BRS Valiosa was derived by backcrossing from Conquista. BRSMG 850G was also found to be very closely related to these two cultivars, which explained the minimal variation among them. The high similarity among these cultivars could explain the difficultly associated with identifying exclusive structural and allelic variations in the genome. In contrast, Santa Rosa, the oldest cultivar used in this study, BRS 284, Doko and IAC 8 have the greatest allelic variation and a large number of structural variations. Because Doko, IAC8 and Santa Rosa are very old cultivars developed in the 1960s and 1970s, the large number of exclusive variations in their genomes could indicate they did not have a large influence on the more recent cultivars.

These findings could be very useful in breeding programs utilizing marker-assisted selection (MAS) and cultivar fingerprinting for cultivar protection. Nonetheless, a validation process will be necessary to confirm the presence of these exclusive alleles in Brazilian cultivars and should be a priority in future studies.

## Conclusions

This is the first genomic analysis of the allelic and structural variations present in Brazilian soybean cultivars. Our results confirmed the hypothesis that the Brazilian soybean germplasm remains narrow. However, it was possible to detect the presence of SNPs and CNVs that distinguished the examined cultivars. The resequencing data allowed the detection of allelic variations that can be applied for identifying genes useful to breeding programs in the future.

Based on our comparison of Brazilian cultivars, we confirmed a large number of allelic modifications in genes associated with the generation of precursor metabolites and energy related to DNA-dependent transcription/elongation and photosynthesis. Such modifications may be related to important functions in the adaptation of soybean to the tropical conditions of Brazil. Furthermore, the presence of a large amount of CNV regions that permit differentiation among the Brazilian germplasm also appears to be a potential target for studies of important agronomic traits. Therefore, further analysis of these CNV regions should be treated as a top priority in the future.

The sub-regions with low diversification identified in Brazilian soybean cultivars may not have been utilized in breeding programs to date. However, these sub-regions may represent targets for the incorporation of new agronomically relevant alleles. In addition, measures to increase the diversity of the Brazilian soybean germplasm should be considered; for example, the use of genotypes from different geographical regions, such as Asian germplasm, or the selection of parental genotypes more divergent for specific genomic regions.

Finally, our resequencing analyses of Brazilian soybean cultivars were able to reveal a large number of exclusive SNPs. These results may constitute an important breeding tool for cultivar fingerprinting and soybean seed protection. However, a validation process will be necessary to confirm our results.

## Methods

### Plant accessions and sequencing

Twenty-eight Brazilian soybean cultivars were selected for this study. The cultivars were selected based on their commercial release date and RMG (Additional file 5: Table S1). These lines were chosen based on their distribution along a 50-year span of the history of soybean breeding in Brazil, consisting of cultivars developed from the 1960s until the present decade. Some of these cultivars were very important as background accessions for modern lines and were cultivated for years in Brazil (e.g., Doko, Santa Rosa, Paraná, FT Cristalina, Conquista, BR 16, Embrapa 48). Moreover, we resequenced some modern elite cultivars (e.g., VMAX RR, NA 5909 RG, BRS 284, BRS Valiosa RR) and others associated with important disease resistance (e.g., BRS/GO Chapadões, the cultivar with resistance to all soybean cyst nematode races). Furthermore, lines from different maturity groups and adapted to different regions of Brazil were also selected. Brazil is located between Ecuador and the Tropic of Capricorn; thus, most of the Brazilian soybean cultivars are located at latitudes 5 to 9 [63]. We also selected lines from South and North Brazil, representing the highest diversity among cultivars.

The seeds were obtained from the germplasm bank of Embrapa Soja or from commercial seed producers. Young leaf tissue samples of each of the 28 Brazilian cultivars were collected at the V3 growth stage. Genomic DNA was isolated from each sample using the Qiagen Mini Plant DNeasy kit (Qiagen Inc., Valencia, CA, USA) following the manufacturer’s instructions. DNA sequencing was performed at FASTERIS Company, Switzerland, using an Illumina Hiseq 2000 platform to generate 100-bp paired-end reads with an expected coverage of 15X of the soybean genome. Sequence data from 19 U.S. soybean lines, which were kindly provided by the Molecular Genetics and Soybean Genomics Laboratory from the University of Missouri, were used for validation.

### SNP and InDel detection

The reads generated by resequencing of the Brazilian soybean accessions were mapped to the new version of the soybean reference genome (Gmax_275_Wm82.a2.v1,https://phytozome.jgi.doe.gov/pz/portal.html) using the alignment program Burrows-Wheeler Aligner (BWA) [64]. After mapping, the aligned reads were processed using Piccard tools version 1.107 to remove duplicate values, and a binary file of the extension bam representing the assembled genome of each resequenced species was generated. For SNP/InDel calling, we used Genome Analysis Toolkit (GATK) version 3.0 [65]. This toolkit was utilized to generate a local realignment in the InDel region and a qualitative recalibration to generate a bam file with fewer errors for each sample. Thus, the new bam files generated were used for SNP/InDel calling of the genome. In both cases, we used the HaplotypeCaller module of GATK.

The analysis was conducted using the bioinformatic NGS resequencing data analysis workflow [66] developed in SoyKB for SNP and Indel calling. XSEDE [67] was used as the computing infrastructure, iPlant as the data and cloud infrastructure [68], and the Pegasus workflow systems [69] to control and coordinate the data management and computational tasks.

### Copy-number variation (CNV) identification

For CNV detection in the soybean genome, we used the Copy-number estimation with a Mixture Of Poissons (cn.MOPS) version 1.10.0 [70]. We also used the SoyKB [71, 72] website to evaluate the presence of modified genes within the detected CNV regions.

### Genetic annotation, functional classification and prediction of important genes

We used the SnpEff program [73] to aid in the functional classification of genes with allelic variations. An enrichment analysis of these modified genes detected through SnpEff was generated using the agriGO [74], SoyBase [16], and SoyKB [71, 72] websites.

### Population structure and diversity analysis

Missing data, deletions and heterozygous SNPs were removed from the dataset. A neighbor-joining phylogenetic tree was constructed using MEGA5 software [75] with the *p-distance* module. A total of 4,938,168 SNPs were used to generate the population structure plot using the FastStructure software [76]. The same numbers of SNPs were used to generate a principal component analysis (PCA) using smartpca program from Eigensoft 4.2 software [77].

For diversity analysis, we estimated the nucleotide diversity within a population (*θπ*) using different sliding windows of different sizes (10 kb, 100 kb and 500 kb) without overlap between adjacent windows. Furthermore, we measured the population fixation index coefficient (F_ST_) using vcftools [78]. For this analysis, we considered the old/oldest cultivars to have been released before 1980 and the newest/latest/modern cultivars after 2000.

### Detection of candidate genes influenced by artificial selection

According to the statistical results obtained in the diversity analysis, we detected some candidate genes influenced by selection. Regions under positive selection tended to have low diversity values and a low allelic frequency between the new and old accessions. The criteria adopted for the region with positive selection were as follows: F_ST_ > 0.45 for the total population distribution and high *θπ* values in the old cultivars. For regions with low diversity, we adopted the criterion of F_ST_ > 0.02. Finally, we used the AgriGO [74], SoyBase [16], and SoyKB [71, 72] websites to generate an enrichment analysis of the genes detected under the influence of positive selection.

## Availability of Supporting Data

All sequence reads described in the manuscript are available at DDBJ/EMBL/GenBank under BioProject accession PRJNA294227. Illumina sequence reads have been deposited at NCBI’s SRA archive under following numbers (SRX1170064, SRX1170065, SRX1170066, SRX1170067, SRX1170068, SRX1170069, SRX1170070, SRX1170071, SRX1170072, SRX1170073, SRX1170074, SRX1170075, SRX1170076, SRX1170077, SRX1170092, SRX1170093, SRX1170094, SRX1170095, SRX1170096, SRX1170953, SRX1170954, SRX1170955, SRX1170956, SRX1170957, SRX1170958, SRX1170959, SRX1170960, SRX1170961). Other supporting data are included as Additional file 1: Figure S1, Additional file 2: Figure S2, Additional file 3: Figure S3, Additional file 4: Figure S4, and Additional file 5: Table S1, Additional file 6: Table S2, Additional file 7: Table S3, Additional file 8: Table S4, Additional file 9: Table S5, and Additional file 10: Table S6.

## Additional files

Additional file 1: Figure S1. Number of homozygous/heterozygous SNPs and InDels for each Brazilian soybean cultivar used in this study. (PNG 456 kb) Additional file 2: Figure S2. Allelic variant analysis of the mapped gene *Dt1* in soybean. **Downstream:** SNPs detected up to 5 kb downstream of the coding region; **Non-synonymous:** SNP variants causing a codon that produces a different amino acid; **Intron:** SNPs detected inside an intron; **Upstream:** SNPs detected up to 5 kb upstream of the coding region. (PNG 50 kb) Additional file 3: Figure S3. Copy number variation for each Brazilian soybean line used in this study. (PNG 144 kb) Additional file 4: Figure S4. Copy number variations detected on Brazilian soybean chromosomes 6, 7, 8, 9,13, 15 and 17. The x-axis represents the genomic position and the y-axis the CNV call produced by the segmentation algorithm. The blue lines are deleted fragments detected in these regions. (PNG 131 kb) Additional file 5: Table S1. Basic description of all Brazilian soybean accessions used in this study. **RMG:** relative maturity group; **Det/Ind:** growing development plant; **Ind:** Indeterminate growing; **Det:** Determinate growing habit. (DOCX 79 kb) Additional file 6: Table S2. Sequencing information for the Brazilian soybean lines (DOCX 95 kb) Additional file 7: Table S3. Variant rate details of the Brazilian soybean accessions. (DOCX 54 kb) Additional file 8: Table S4. Number of SNPs associated to important regions on Brazilian soybean cultivars. **All:** SNP present in all Brazilian cultivars compared to reference genome; **Non-syn cds:** non-synonymous SNP inside coding region, **Start G.:** A variant in 5'UTR region produces a three base sequence that can be a START codon; **Start L.:** Variant causes start codon to be mutated into a non-start codon; **Stop G.:** Variant causes a STOP codon; **Stop L.:** Variant causes stop codon to be mutated into a non-stop codon; **Splice Site A.:** The variant hits a splice acceptor site; **Splice Site D.:** The variant hits a Splice donor site. (DOCX 109 kb) Additional file 9: Table S5. Summary of the most relevant results from the Gene Ontology (GO) enrichment analysis. (DOCX 117 kb) Additional file 10: Table S6. Number of non-synonymous InDels identified in important regions of the Brazilian soybean cultivars. **All:** SNP present in all Brazilian cultivars compared to reference genome; **Disruptive + Inframe Del:** one codon is changed and one or more codons are deleted; **Disruptive + Inframe Ins:** one codon is changed and one or many codons are inserted; **Inframe Del:** one or many codons are deleted; **Inframe Ins:** one or many codons are inserted; **Frame var:** insertion or deletion causes a frame shift; **Exon loss:** a deletion removes the whole exon; **Start lost:** Variant causes start codon to be mutated into a non-start codon; **Stop G.:** Variant causes a STOP codon; **Stop L.:** Variant causes stop codon to be mutated into a non-stop codon; **Splice Site A.:** The variant hits a splice acceptor site; **Splice Site D.:** The variant hits a Splice donor site. (DOCX 131 kb)

## Abbreviations

SNPs: single nucleotide polymorphisms
CNVs: copy-number variations
cv.: cultivar
GWAS: genome-wide association analysis
Mb: megabase
kb: kilbase
ts/tv: transition/transversion
RMG: Relative Maturity Group
NJ: neighbor-joining
PCA: principal component analysis
MAS: marker-assisted selection
BWA: Burrows-Wheeler Aligner
GATK: Genome Analysis Toolkit
GO: Gene Ontology
