# Supplementary figures and images for: Evaluation of genetic variation among Brazilian soybean cultivars through genome resequencing
Source: BMC Genomics. 2016 Feb 13;17:110. doi: 10.1186/s12864-016-2431-x (PMC4752768; doi:10.1186/s12864-016-2431-x)

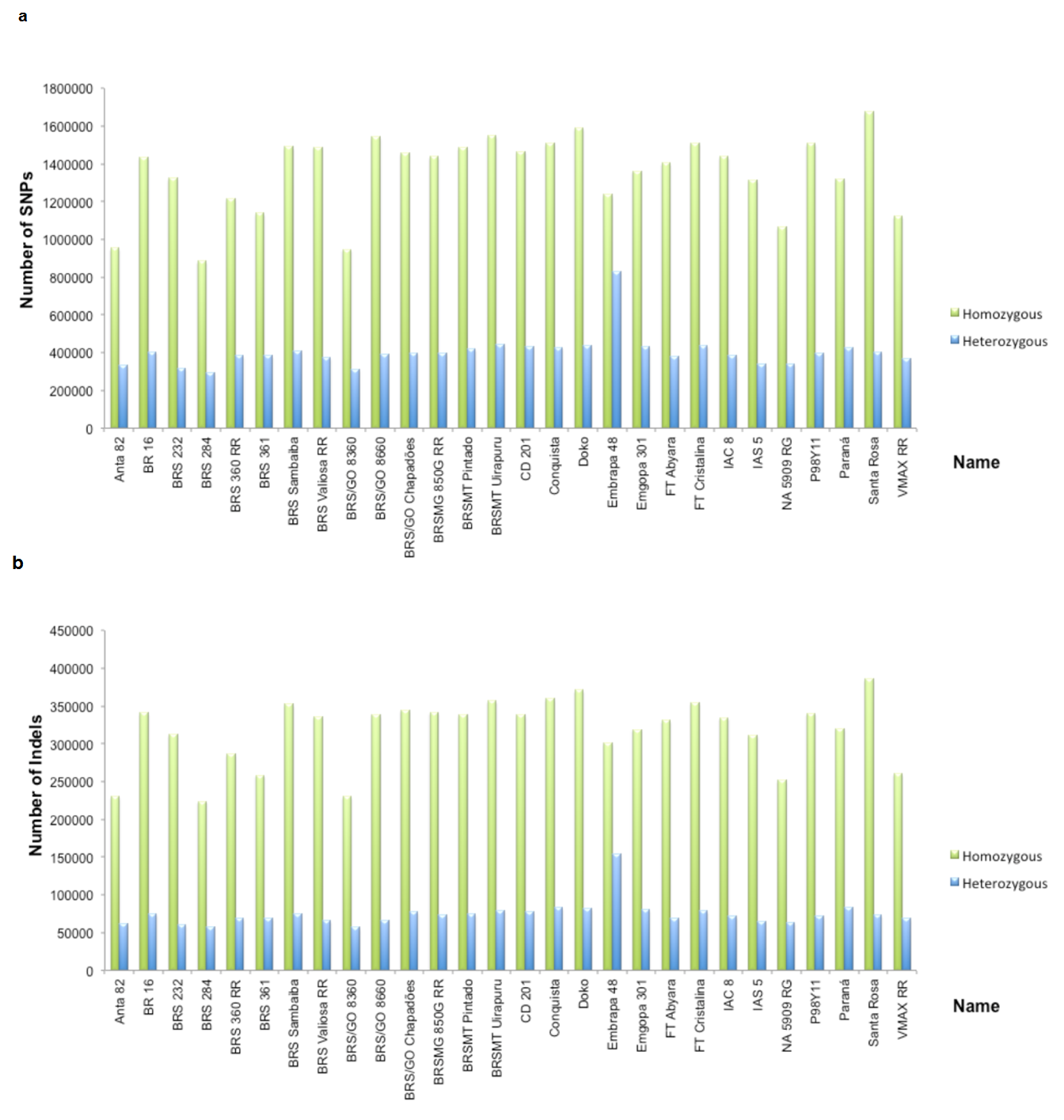

Supplement: Additional file 1: Figure S1. — Number of homozygous/heterozygous SNPs and InDels for each Brazilian soybean cultivar used in this study. (PNG 456 kb) [file 12864_2016_2431_MOESM1_ESM.png]

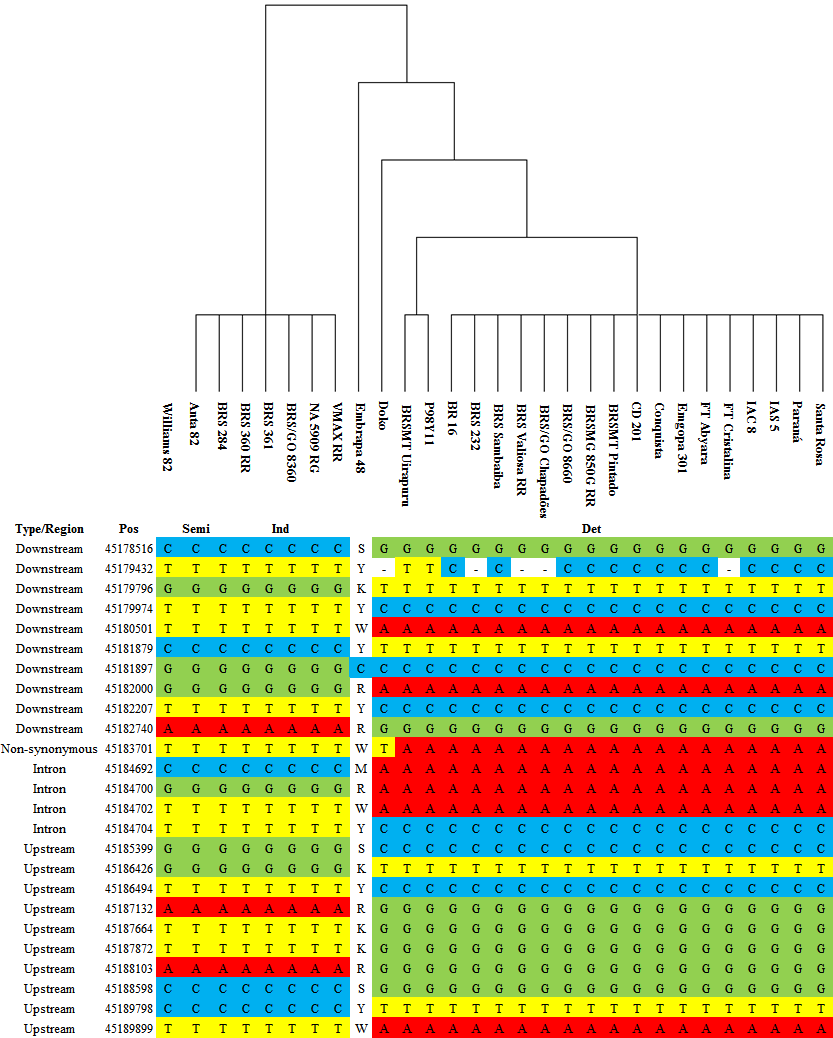

Supplement: Additional file 2: Figure S2. — Allelic variant analysis of the mapped gene Dt1 in soybean. Downstream: SNPs detected up to 5 kb downstream of the coding region; Non-synonymous: SNP variants causing a codon that produces a different amino acid; Intron: SNPs detected inside an intron; Upstream: SNPs detected up to 5 kb upstream of the coding region. (PNG 50 kb) [file 12864_2016_2431_MOESM2_ESM.png]

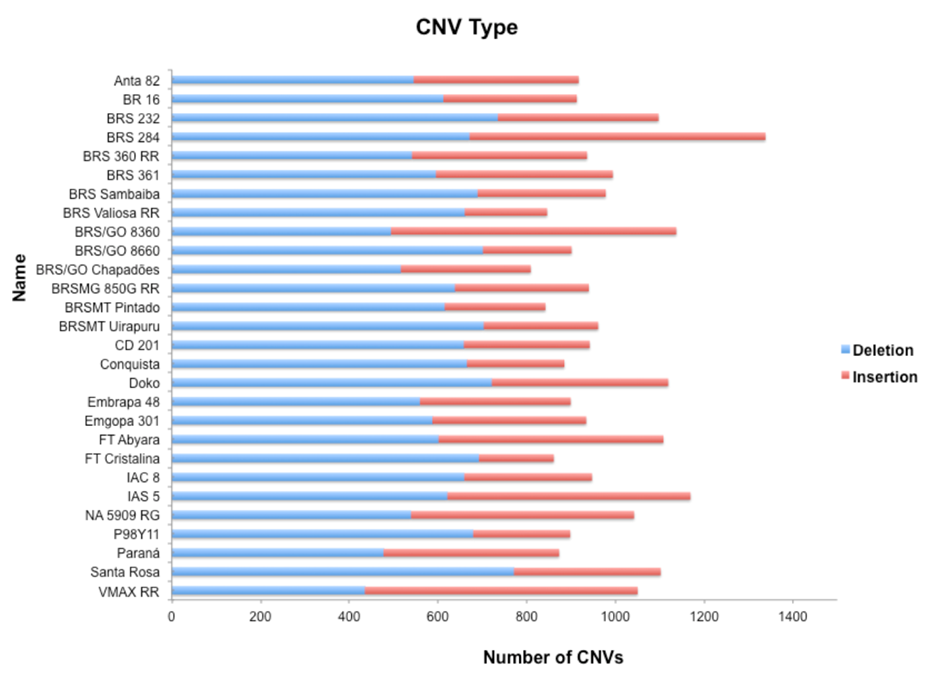

Supplement: Additional file 3: Figure S3. — Copy number variation for each Brazilian soybean line used in this study. (PNG 144 kb) [file 12864_2016_2431_MOESM3_ESM.png]

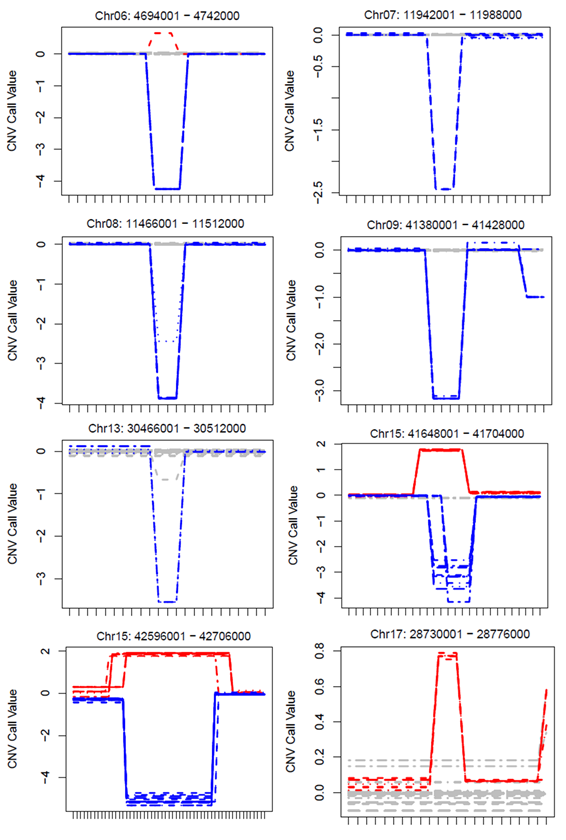

Supplement: Additional file 4: Figure S4. — Copy number variations detected on Brazilian soybean chromosomes 6, 7, 8, 9,13, 15 and 17. The x-axis represents the genomic position and the y-axis the CNV call produced by the segmentation algorithm. The blue lines are deleted fragments detected in these regions. (PNG 131 kb) [file 12864_2016_2431_MOESM4_ESM.png]
