# Supplementary material for: Evaluation of genetic variation among Brazilian soybean cultivars through genome resequencing
Source: BMC Genomics. 2016 Feb 13;17:110. doi: 10.1186/s12864-016-2431-x (PMC4752768; doi:10.1186/s12864-016-2431-x)
Supplement: Additional file 5: Table S1. — Basic description of all Brazilian soybean accessions used in this study. RMG: relative maturity group; Det/Ind: growing development plant; Ind: Indeterminate growing; Det: Determinate growing habit. (DOCX 79 kb) [file 12864_2016_2431_MOESM5_ESM.docx]

**Additional Table 1.** Basic description of all Brazilian soybean accessions used in this study.

| **Access Name** | **Decade** | **Maturity Group** | **Det/Indet** |
| --- | --- | --- | --- |
| **Santa Rosa** | 1961-1970 | 7.5-7.8 | Determinate |
| **IAC 8** | 1971-1980 | 8.0 | Determinate |
| **IAS 5** | 1971-1980 | 6.4 | Determinate |
| **Paraná** | 1971-1980 | 6.7-6.9 | Determinate |
| **Doko** | 1981-1990 | 9.0 | Determinate |
| **EMGOPA 301** | 1981-1990 | 8.0 | Determinate |
| **FT Abyara** | 1981-1990 | 7.3-7.6 | Determinate |
| **FT Cristalina** | 1981-1990 | 7.6 | Determinate |
| **BR 16** | 1991-2000 | 6.4 | Determinate |
| **BRSMT Pintado** | 1991-2000 | 8.4 | Determinate |
| **BRSMT Uirapuru** | 1991-2000 | 9.0 | Determinate |
| **CD 201** | 1991-2000 | 6.6 | Determinate |
| **Embrapa 48** | 1991-2000 | 6.8 | Determinate |
| **MG/BR46** | 1991-2000 | 8.1 | Determinate |
| **Anta 82** | 2001-2010 | 7.2 | Semideterminate |
| **BRS 232** | 2001-2010 | 6.9 | Determinate |
| **BRS 284** | 2001-2010 | 6.3 | Indeterminate |
| **BRS Sambaíba** | 2001-2010 | 9.3 | Determinate |
| **BRS Valiosa RR** | 2001-2010 | 8.1 | Determinate |
| **BRSGO 8360** | 2001-2010 | 8.1 | Indeterminate |
| **BRSGO 8660** | 2001-2010 | 8.6 | Determinate |
| **BRSGO Chapadões** | 2001-2010 | 8.6 | Determinate |
| **BRSMG 850 GRR** | 2001-2010 | 8.2 | Determinate |
| **NA 5909 RG** | 2001-2010 | 6.2 | Indeterminate |
| **P98Y11** | 2001-2010 | 8.1 | Determinate |
| **V MAX RR** | 2001-2010 | 6.2 | Indeterminate |
| **BRS 360RR** | 2011-2020 | 6.2 | Indeterminate |
| **BRS 361** | 2011-2020 | 7.4 | Indeterminate |

**RMG:** relative maturity group; **Det/Ind:** growing development plant; **Ind:** Indeterminate growing; **Det:** Determinate growing habit.
