# Supplementary material for: Evaluation of genetic variation among Brazilian soybean cultivars through genome resequencing
Source: BMC Genomics. 2016 Feb 13;17:110. doi: 10.1186/s12864-016-2431-x (PMC4752768; doi:10.1186/s12864-016-2431-x)
Supplement: Additional file 6: Table S2. — Sequencing information for the Brazilian soybean lines (DOCX 95 kb) [file 12864_2016_2431_MOESM6_ESM.docx]

**Additional Table 2.** Sequencing information for the Brazilian soybean lines.

| **Accession Name** | **Number of reads** | **Number of mapped reads** | **Genome coverage** | **Mean depth** |
| --- | --- | --- | --- | --- |
| **Anta 82** | 195,886,570 | 180,622,083 | 0.9221 | 13.2579 |
| **BR 16** | 196,397,054 | 186,322,940 | 0.9487 | 14.7765 |
| **BRS 232** | 122,368,531 | 116,984,169 | 0.9560 | 9.1884 |
| **BRS 284** | 171,461,982 | 163,470,705 | 0.9534 | 12.6584 |
| **BRS 360RR** | 201,402,004 | 192,681,470 | 0.9567 | 15.3996 |
| **BRS 361** | 191,425,924 | 183,136,909 | 0.9567 | 14.6088 |
| **BRS Sambaíba** | 192,188,713 | 181,426,603 | 0.9440 | 14.7690 |
| **BRS Valiosa RR** | 156,259,988 | 148,106,406 | 0.9478 | 11.4630 |
| **BRSGO 8360** | 158,327,168 | 148,939,413 | 0.9407 | 11.1166 |
| **BRSGO 8660** | 142,045,602 | 133,525,390 | 0.9400 | 10.5473 |
| **BRSGO Chapadões** | 245,514,627 | 222,295,561 | 0.9054 | 17.6885 |
| **BRSMG 850 GRR** | 217,891,058 | 206,849,339 | 0.9493 | 16.4315 |
| **BRSMT Pintado** | 191,564,772 | 181,730,846 | 0.9487 | 14.4552 |
| **BRSMT Uirapuru** | 198,605,106 | 186,387,017 | 0.9385 | 15.2175 |
| **CD 201** | 221,562,422 | 210,956,658 | 0.9521 | 17.2033 |
| **Doko** | 247,331,922 | 234,203,818 | 0.9469 | 19.2339 |
| **Embrapa 48** | 211,246,973 | 195,673,675 | 0.9263 | 15.9426 |
| **EMGOPA 301** | 159,411,439 | 150,539,582 | 0.9443 | 12.1211 |
| **FT Abyara** | 185,414,289 | 175,454,862 | 0.9463 | 13.9882 |
| **FT Cristalina** | 223,191,658 | 213,329,190 | 0.9558 | 17.6043 |
| **IAC 8** | 204,515,975 | 194,693,512 | 0.9520 | 15.7266 |
| **IAS 5** | 150,707,921 | 133,763,689 | 0.8876 | 10.8346 |
| **MG/BR46** | 272,124,638 | 258,659,654 | 0.9505 | 21.4019 |
| **NA 5909 RG** | 189,097,657 | 179,353,466 | 0.9485 | 13.8281 |
| **P98Y11** | 149,136,838 | 141,851,602 | 0.9512 | 11.2006 |
| **Paraná** | 317,943,922 | 301,620,197 | 0.9487 | 23.9907 |
| **Santa Rosa** | 166,171,639 | 157,805,234 | 0.9497 | 12.6687 |
| **V MAX RR** | 221,332,482 | 207,971,094 | 0.9396 | 16.3037 |
