# Supplementary material for: Evaluation of genetic variation among Brazilian soybean cultivars through genome resequencing
Source: BMC Genomics. 2016 Feb 13;17:110. doi: 10.1186/s12864-016-2431-x (PMC4752768; doi:10.1186/s12864-016-2431-x)
Supplement: Additional file 7: Table S3. — Variant rate details of the Brazilian soybean accessions. (DOCX 54 kb) [file 12864_2016_2431_MOESM7_ESM.docx]

**Additional Table 3.** Variant rate details for the Brazilian soybean accessions.

| **Chromosome** | **Length** | **Number of SNPs** | **Variants rate** |
| --- | --- | --- | --- |
| 1 | 56,831,624 | 263,927 | 215 |
| 2 | 48,577,505 | 273,274 | 177 |
| 3 | 45,779,781 | 317,779 | 144 |
| 4 | 52,389,146 | 313,989 | 166 |
| 5 | 42,234,498 | 182,558 | 231 |
| 6 | 51,416,486 | 330,362 | 155 |
| 7 | 44,630,646 | 241,445 | 184 |
| 8 | 47,837,940 | 240,096 | 199 |
| 9 | 50,189,764 | 306,025 | 164 |
| 10 | 51,566,898 | 223,063 | 231 |
| 11 | 34,766,867 | 169,064 | 205 |
| 12 | 40,091,314 | 190,138 | 210 |
| 13 | 45,874,162 | 315,257 | 145 |
| 14 | 49,042,192 | 333,541 | 147 |
| 15 | 51,756,343 | 477,983 | 108 |
| 16 | 37,887,014 | 318,763 | 118 |
| 17 | 41,641,366 | 253,241 | 164 |
| 18 | 58,018,742 | 541,951 | 107 |
| 19 | 50,746,916 | 312,722 | 162 |
| 20 | 47,904,181 | 263,166 | 182 |
| **Total** | **949,183,385** | **5,868,344** | **161** |
