# Supplementary material for: Evaluation of genetic variation among Brazilian soybean cultivars through genome resequencing
Source: BMC Genomics. 2016 Feb 13;17:110. doi: 10.1186/s12864-016-2431-x (PMC4752768; doi:10.1186/s12864-016-2431-x)
Supplement: Additional file 8: Table S4. — Number of SNPs associated to important regions on Brazilian soybean cultivars. All: SNP present in all Brazilian cultivars compared to reference genome; Non-syn cds: non-synonymous SNP inside coding region, Start G.: A variant in 5'UTR region produces a three base sequence that can be a START codon; Start L.: Variant causes start codon to be mutated into a non-start codon; Stop G.: Variant causes a STOP codon; Stop L.: Variant causes stop codon to be mutated into a non-stop codon; Splice Site A.: The variant hits a splice acceptor site; Splice Site D.: The variant hits a Splice donor site. (DOCX 109 kb) [file 12864_2016_2431_MOESM8_ESM.docx]

**Additional Table 4.** Number of SNPs associated with important regions of the Brazilian soybean cultivars.

| **Cultivars** | **Modifications** | | | | | | | **Total** |
| --- | --- | --- | --- | --- | --- | --- | --- | --- |
|  | **Non-syn cds** | **Start** | | **Stop** | | **Splice Site** | |  |
|  |  | **G.** | **L.** | **G.** | **L.** | **A.** | **D.** |  |
| **All** | 551 | 40 | 8 | 2 | 4 | 0 | 4 | 609 |
| **Anta 82** | 17,645 | 1,188 | 57 | 351 | 89 | 135 | 125 | 19,590 |
| **BR 16** | 27,196 | 1,868 | 76 | 511 | 130 | 187 | 174 | 30,142 |
| **BRS 232** | 25,390 | 1,730 | 71 | 498 | 124 | 171 | 176 | 28,160 |
| **BRS 284** | 17,708 | 1,197 | 47 | 345 | 100 | 120 | 113 | 19,630 |
| **BRS 360 RR** | 22,094 | 1,465 | 71 | 429 | 107 | 166 | 159 | 24,491 |
| **BRS 361** | 19,716 | 1,301 | 65 | 390 | 106 | 153 | 127 | 21,858 |
| **BRS Sambaiba** | 28,275 | 1,908 | 84 | 576 | 133 | 216 | 185 | 31,377 |
| **BRS Valiosa RR** | 26,835 | 1,833 | 96 | 529 | 124 | 191 | 168 | 29,776 |
| **BRSGO 8360** | 18,184 | 1,209 | 49 | 380 | 99 | 123 | 121 | 20,165 |
| **BRSGO 8660** | 27,343 | 1,851 | 80 | 562 | 137 | 206 | 188 | 30,367 |
| **BRSGO Chapadões** | 26,580 | 1,707 | 81 | 517 | 115 | 212 | 170 | 29,382 |
| **BRSMG 850G RR** | 26,646 | 1,826 | 93 | 517 | 127 | 195 | 160 | 29,564 |
| **BRSMT Pintado** | 25,638 | 1,699 | 77 | 509 | 127 | 178 | 173 | 28,401 |
| **BRSMT Uirapuru** | 27,922 | 1,882 | 68 | 555 | 154 | 193 | 207 | 30,981 |
| **CD 201** | 25,908 | 1,733 | 77 | 509 | 121 | 213 | 185 | 28,746 |
| **Conquista** | 27,083 | 1,863 | 97 | 550 | 125 | 192 | 162 | 30,072 |
| **Doko** | 28,709 | 1,988 | 91 | 569 | 138 | 204 | 180 | 31,879 |
| **Embrapa 48** | 23,984 | 1,539 | 68 | 440 | 114 | 165 | 156 | 26,466 |
| **Emgopa 301** | 24,929 | 1,726 | 71 | 516 | 116 | 180 | 187 | 27,725 |
| **FT Abyara** | 26,400 | 1,765 | 82 | 516 | 140 | 204 | 166 | 29,273 |
| **FT Cristalina** | 27,593 | 1,874 | 70 | 551 | 148 | 193 | 207 | 30,636 |
| **IAC 8** | 26,043 | 1,752 | 87 | 543 | 118 | 174 | 151 | 28,868 |
| **IAS 5** | 24,266 | 1,681 | 66 | 475 | 122 | 177 | 169 | 26,956 |
| **NA 5909 RG** | 19,150 | 1,241 | 65 | 356 | 87 | 151 | 136 | 21,186 |
| **P98Y11** | 27,082 | 1,811 | 84 | 536 | 132 | 203 | 176 | 30,024 |
| **Paraná** | 24,322 | 1,676 | 67 | 479 | 123 | 172 | 163 | 27,002 |
| **Santa Rosa** | 30,217 | 2,042 | 98 | 634 | 152 | 220 | 208 | 33,571 |
| **VMAX RR** | 18,936 | 1,241 | 69 | 378 | 85 | 154 | 137 | 21,000 |

**All:** SNP present in all Brazilian cultivars compared to reference genome; **Non-syn cds:** non-synonymous SNP inside coding region, **Start G.:** A variant in 5'UTR region produces a three base sequence that can be a START codon; **Start L.:** Variant causes start codon to be mutated into a non-start codon; **Stop G.:** Variant causes a STOP codon; **Stop L.:** Variant causes stop codon to be mutated into a non-stop codon; **Splice Site A.:** The variant hits a splice acceptor site; **Splice Site D.:** The variant hits a Splice donor site.
