# Supplementary material for: Evaluation of genetic variation among Brazilian soybean cultivars through genome resequencing
Source: BMC Genomics. 2016 Feb 13;17:110. doi: 10.1186/s12864-016-2431-x (PMC4752768; doi:10.1186/s12864-016-2431-x)
Supplement: Additional file 9: Table S5. — Summary of the most relevant results from the Gene Ontology (GO) enrichment analysis. (DOCX 117 kb) [file 12864_2016_2431_MOESM9_ESM.docx]

**Additional Table 5.** Summary of the most relevant results from the GO enrichment analysis.

| **Description** | **Number of genes** | **Genes** |
| --- | --- | --- |
| Generation of precursor metabolites and energy | 34 | Glyma.01G058600, Glyma.01G076000, Glyma.01G091900, Glyma.01G095900, Glyma.01G153500, Glyma.01G201600, Glyma.04G095000, Glyma.04G204000, Glyma.05G073600, Glyma.06G217900, Glyma.07G143800, Glyma.08G281300, Glyma.09G171300, Glyma.09G178600, Glyma.10G226400, Glyma.11G114700, Glyma.12G056400, Glyma.13G088500, Glyma.13G155500, Glyma.14G149900, Glyma.15G114600, Glyma.15G188400, Glyma.15G239000, Glyma.15G238900, Glyma.18G078500, Glyma.18G155300, Glyma.18G155400, Glyma.18G203700, Glyma.19G051900, Glyma.19G053400, Glyma.19G054200, Glyma.19G081000, Glyma.19G083500, Glyma.19G109600 |
| DNA-dependent transcription, elongation | 34 | Glyma.01G058600, Glyma.01G076000, Glyma.01G091900, Glyma.01G095900, Glyma.01G153500, Glyma.01G201600, Glyma.04G095000, Glyma.04G204000, Glyma.05G073600, Glyma.06G217900, Glyma.07G143800, Glyma.08G281300, Glyma.09G171300, Glyma.09G178600, Glyma.10G226400, Glyma.11G114700, Glyma.12G056400, Glyma.13G088500, Glyma.13G155500, Glyma.14G149900, Glyma.15G114600, Glyma.15G188400, Glyma.15G239000, Glyma.15G238900, Glyma.18G078500, Glyma.18G155300, Glyma.18G155400, Glyma.18G203700, Glyma.19G051900, Glyma.19G053400, Glyma.19G054200, Glyma.19G081000, Glyma.19G083500, Glyma.19G109600 |
| Photosynthesis | 38 | Glyma.01G058600, Glyma.01G076000, Glyma.01G091900, Glyma.01G095900, Glyma.01G153500, Glyma.01G201600, Glyma.04G095000, Glyma.04G204000, Glyma.05G073600, Glyma.06G217900, Glyma.06G228400, Glyma.07G143800, Glyma.08G281300, Glyma.09G171300, Glyma.09G178600, Glyma.10G226400, Glyma.11G081100, Glyma.11G114700, Glyma.12G056400, Glyma.13G088500, Glyma.13G155500, Glyma.14G149900, Glyma.15G114600, Glyma.15G188400, Glyma.15G239000, Glyma.15G238900, Glyma.15G246000, Glyma.18G078500, Glyma.18G155300, Glyma.18G155400, Glyma.18G203700, Glyma.18G262700, Glyma.19G051900, Glyma.19G053400, Glyma.19G054200, Glyma.19G081000, Glyma.19G083500, Glyma.19G109600 |
| Photosynthesis, light reaction | 19 | Glyma.01G058600, Glyma.01G095900, Glyma.01G153500, Glyma.04G095000, Glyma.05G073600, Glyma.06G217900, Glyma.07G143800, Glyma.07G201300, Glyma.08G281300, Glyma.11G114700, Glyma.12G056400, Glyma.13G088500, Glyma.15G114600, Glyma.18G155300, Glyma.18G155400, Glyma.19G053400, Glyma.19G081000, Glyma.19G083500, Glyma.19G109600 |
| ATP synthesis coupled electron transport | 6 | Glyma.01G101600, Glyma.06G228400, Glyma.10G068800, Glyma.11G081100, Glyma.15G246000, Glyma.18G155400 |
| Photosynthetic electron transport in photosystem II | 6 | Glyma.01G153500, Glyma.04G095000, Glyma.05G073600, Glyma.06G217900, Glyma.11G114700, Glyma.18G262700 |
| Cellular respiration | 7 | Glyma.06G228400, Glyma.11G081100, Glyma.12G056400, Glyma.15G246000, Glyma.18G262700, Glyma.19G081000, Glyma.19G083500 |
