# Supplementary material for: Evaluation of genetic variation among Brazilian soybean cultivars through genome resequencing
Source: BMC Genomics. 2016 Feb 13;17:110. doi: 10.1186/s12864-016-2431-x (PMC4752768; doi:10.1186/s12864-016-2431-x)
Supplement: Additional file 10: Table S6. — Number of non-synonymous InDels identified in important regions of the Brazilian soybean cultivars. All: SNP present in all Brazilian cultivars compared to reference genome; Disruptive + Inframe Del: one codon is changed and one or more codons are deleted; Disruptive + Inframe Ins: one codon is changed and one or many codons are inserted; Inframe Del: one or many codons are deleted; Inframe Ins: one or many codons are inserted; Frame var: insertion or deletion causes a frame shift; Exon loss: a deletion removes the whole exon; Start lost: Variant causes start codon to be mutated into a non-start codon; Stop G.: Variant causes a STOP codon; Stop L.: Variant causes stop codon to be mutated into a non-stop codon; Splice Site A.: The variant hits a splice acceptor site; Splice Site D.: The variant hits a Splice donor site. (DOCX 131 kb) [file 12864_2016_2431_MOESM10_ESM.docx]

**Additional Table 6.** Number of non-synonymous InDels identified in important regions of the Brazilian soybean cultivars.

| **Cultivars** | **Modifications** | | | | | | | | | | | **Total** |
| --- | --- | --- | --- | --- | --- | --- | --- | --- | --- | --- | --- | --- |
|  | **Disruptive + Inframe** | | **Inframe** | | **Frame var** | **Exon loss** | **Start lost** | **Stop** | | **Splice Site** | |  |
|  | **Del** | **Ins** | **Del** | **Ins** |  |  |  | **G.** | **L.** | **A.** | **D.** |  |
| **All** | 5 | 1 | 1 | 6 | 45 | 0 | 0 | 0 | 1 | 0 | 0 | 59 |
| **Anta 82** | 190 | 108 | 496 | 463 | 2,143 | 0 | 42 | 48 | 44 | 72 | 73 | 3,679 |
| **BR 16** | 283 | 171 | 796 | 738 | 2,876 | 1 | 58 | 82 | 51 | 89 | 119 | 5,264 |
| **BRS 232** | 242 | 152 | 688 | 663 | 2,609 | 0 | 55 | 70 | 53 | 81 | 86 | 4,699 |
| **BRS 284** | 200 | 121 | 508 | 483 | 2,148 | 0 | 41 | 53 | 41 | 64 | 82 | 3,741 |
| **BRS 360 RR** | 234 | 140 | 613 | 608 | 2,493 | 0 | 51 | 62 | 52 | 77 | 87 | 4,417 |
| **BRS 361** | 201 | 112 | 562 | 455 | 2,303 | 0 | 41 | 51 | 43 | 75 | 89 | 3,932 |
| **BRS Sambaiba** | 275 | 180 | 786 | 809 | 2,904 | 1 | 54 | 74 | 56 | 97 | 113 | 5,349 |
| **BRS Valiosa RR** | 274 | 166 | 759 | 723 | 2,781 | 0 | 48 | 83 | 56 | 86 | 112 | 5,088 |
| **BRSGO 8360** | 188 | 123 | 521 | 544 | 2,286 | 0 | 47 | 53 | 37 | 65 | 78 | 3,942 |
| **BRSGO 8660** | 269 | 153 | 745 | 713 | 2,726 | 0 | 50 | 72 | 47 | 92 | 84 | 4,951 |
| **BRSGO Chapadões** | 265 | 172 | 741 | 769 | 2,879 | 0 | 51 | 79 | 56 | 85 | 106 | 5,203 |
| **BRSMG 850G RR** | 286 | 172 | 784 | 747 | 2,881 | 0 | 50 | 85 | 52 | 86 | 104 | 5,247 |
| **BRSMT Pintado** | 277 | 161 | 722 | 703 | 2,736 | 0 | 61 | 68 | 53 | 81 | 102 | 4,964 |
| **BRSMT Uirapuru** | 310 | 181 | 815 | 773 | 2,947 | 0 | 62 | 74 | 54 | 97 | 110 | 5,423 |
| **CD 201** | 291 | 156 | 774 | 691 | 2,804 | 1 | 60 | 73 | 54 | 94 | 102 | 5,100 |
| **Conquista** | 285 | 178 | 788 | 780 | 2,937 | 0 | 54 | 100 | 60 | 90 | 116 | 5,388 |
| **Doko** | 294 | 162 | 829 | 773 | 2,962 | 0 | 59 | 87 | 55 | 99 | 105 | 5,425 |
| **Embrapa 48** | 240 | 164 | 682 | 689 | 2,655 | 1 | 49 | 63 | 53 | 85 | 102 | 4,783 |
| **Emgopa 301** | 251 | 171 | 712 | 700 | 2,716 | 1 | 55 | 72 | 54 | 91 | 93 | 4,916 |
| **FT Abyara** | 273 | 186 | 723 | 763 | 2,796 | 0 | 59 | 79 | 57 | 91 | 97 | 5,124 |
| **FT Cristalina** | 303 | 174 | 804 | 769 | 2,952 | 0 | 60 | 77 | 58 | 88 | 107 | 5,392 |
| **IAC 8** | 257 | 167 | 732 | 727 | 2,791 | 0 | 54 | 74 | 56 | 87 | 109 | 5,054 |
| **IAS 5** | 255 | 145 | 651 | 647 | 2,446 | 0 | 56 | 68 | 45 | 84 | 90 | 4,487 |
| **NA 5909 RG** | 209 | 128 | 553 | 539 | 2,345 | 0 | 44 | 62 | 45 | 75 | 71 | 4,071 |
| **P98Y11** | 292 | 167 | 779 | 734 | 2,768 | 0 | 58 | 79 | 53 | 82 | 103 | 5,115 |
| **Paraná** | 263 | 155 | 707 | 679 | 2,686 | 0 | 50 | 75 | 47 | 87 | 88 | 4,837 |
| **Santa Rosa** | 305 | 193 | 855 | 815 | 2,995 | 0 | 62 | 83 | 51 | 101 | 107 | 5,567 |
| **VMAX RR** | 202 | 121 | 554 | 509 | 2,345 | 0 | 40 | 57 | 50 | 77 | 86 | 4,041 |

**All:** SNP present in all Brazilian cultivars compared to reference genome; **Disruptive + Inframe Del:** one codon is changed and one or more codons are deleted; **Disruptive + Inframe Ins:** one codon is changed and one or many codons are inserted; **Inframe Del:** one or many codons are deleted; **Inframe Ins:** one or many codons are inserted; **Frame var:** insertion or deletion causes a frame shift; **Exon loss:** a deletion removes the whole exon; **Start lost:** Variant causes start codon to be mutated into a non-start codon; **Stop G.:** Variant causes a STOP codon; **Stop L.:** Variant causes stop codon to be mutated into a non-stop codon; **Splice Site A.:** The variant hits a splice acceptor site; **Splice Site D.:** The variant hits a Splice donor site.
